# Long-term metabolic and cardiovascular health benefits from romantic partner touch in older adults

**DOI:** 10.1016/j.bbih.2026.101352

**Published:** 2026-09-09

**Authors:** Gabriele Navyte, Helge Gillmeister, Meena Kumari

**Affiliations:** a Institute for Social and Economic Research, University of Essex, Wivenhoe Park, Colchester, CO4 3SQ, United Kingdom; b Department of Psychology, University of Essex, Wivenhoe Park, Colchester, CO4 3SQ, United Kingdom

**Keywords:** Interpersonal touch, Older adults, Romantic partners, Allostatic load, *Metabolic health*, *Cardiovascular health*, Neuroendocrine health, Longitudinal associations

## Abstract

Interpersonal touch is increasingly recognised as a health-relevant form of social support, associated with improved well-being and stress regulation, with emerging evidence suggesting longer-term physiological associations. Most research on touch has focused on cross-sectional associations of single health markers in young adults, leaving gaps in understanding the association of interpersonal touch with multisystem health over time, especially in groups at risk of low exposure. Chronic stress can have cumulative multiphysiological effects, for example, allostatic load. This study investigates associations between touch frequency with romantic partners and other close adults and components of allostatic load derived from factor analysis, representing three domains: metabolic (C-reactive protein, glycosylated haemoglobin, body mass index, and waist circumference), cardiovascular (systolic blood pressure and diastolic blood pressure), and neuroendocrine (dehydroepiandrosterone and pulse) health measured at two time points five years apart in older adults. Data were drawn from the National Social Life, Health, and Aging Project (n = 787; mean age 73.34y). Cross-lagged path models assessed the relationships between touch frequency and health outcomes, while adjusting for relevant covariates. Frequent touch with romantic partners was associated with more favourable metabolic (β = −0.08, *p* = .006) and cardiovascular (β = −0.14, *p* = .005) health profiles five years later. However, touch frequency with other close adults was not associated with improvements in these health domains. These findings highlight a unique and sustained association between frequent romantic partner touch and metabolic and cardiovascular health, suggesting that regular touch may be linked to lower chronic stress-related physiological burden over time.

## Highlights

• Cross-lagged path models assessed touch frequency and allostatic load over time.
• Touch with partners, not other adults, predicts lower physiological stress markers.
• Frequent partner touch predicts better older adult metabolic health 5 years later.
• Frequent partner touch is linked to improved long-term cardiovascular health.
• Regular touch may mitigate chronic stress and its physiological burden over time.

## 1 Introduction

Social support is defined as the perception or experience that one is loved and cared for by others, esteemed and valued, and part of a social network of mutual assistance and obligations (Wills, 1991). It is a well-established determinant of health, associated with improved psychological outcomes and reduced morbidity and mortality (Yang et al., 2016; and see reviews by Berkman et al., 2000; Cohen, 1988; Coleman and Iso-Ahola, 1993; House et al., 1988; Seeman, 1996; Uchino, 2004, 2006). Although often studied through verbal communication, social support can also be conveyed through nonverbal behaviours (Robinson et al., 2015). Interpersonal touch is a particularly salient form of nonverbal social interaction and has been proposed as one pathway through which social support may be linked to health (Che et al., 2018; Dreisoerner et al., 2021; Morrison, 2016; Roberts et al., 2015; Schneider et al., 2023).

Touch behaviours such as stroking, hand-holding, and hugging convey proximity and affiliation, signals that are often interpreted as cues of safety (Eckstein et al., 2020). This is thought to be mediated partly by oxytocin release, which enhances bonding while attenuating stress responses (Kreuder et al., 2019; Carter, 1998; Tang et al., 2020; Uvnäs-Moberg, 1998). Complementary evidence implicates increased vagal activity during touch, supporting parasympathetic dominance and reducing activation of both the hypothalamic-pituitary-adrenal (HPA) axis and the autonomic nervous system (ANS) (Ferrell-Torry and Glick, 1993; Field, 1998, 2010; Lindgren et al., 2010; Shaltout et al., 2012; Van Puyvelde et al., 2021). This aligns with the innate drive to seek comfort, which underpins early attachment formation (Bowlby, 1982; Harlow, 1958), and serves a core regulatory function in stress attenuation. Physical touch is a primary means through which this regulation is achieved. Evidence from experimental studies suggests that specific forms of touch can influence acute stress responses in adults. In a laboratory functional magnetic resonance imaging (fMRI) study, hand-holding from a spouse attenuated neural responses to threat compared with stranger hand-holding or no touch (Coan et al., 2006). Similarly, a randomised intervention study found that couples trained to engage in regular touch (on the neck, shoulder, and hand) for four weeks showed reduced salivary alpha-amylase, a marker of sympathetic activity, relative to a monitoring-only control group (Holt-Lunstad et al., 2008). A further randomised experimental study found that brief partner-delivered neck and shoulder massage before an acute stressor reduced cortisol responses and attenuated heart rate increases compared with verbal support or no interaction (Ditzen et al., 2007). Thus, these findings position touch as a possible pathway linking social support to stress regulation.

Stress, broadly defined as the response to perceived threats to psychological or physiological integrity, mobilises the body's regulatory networks to enable short-term adaptation (McEwen, 2013). Activation of the sympathetic nervous system releases adrenaline to prepare immediate action, while the HPA axis releases cortisol and other hormones to sustain activity (Herman et al., 2020; McEwen and Akil, 2020). These processes are adaptive in the short term but carry costs when repeatedly or chronically activated. The concept of allostasis captures this dynamic regulation, whereby the body maintains stability through change (Sterling and Eyer, 1988; McEwen, 1998). When stress responses are prolonged, the resulting allostatic load imposes wear and tear on physiological systems, increasing vulnerability to disease across multiple organ systems (McEwen, 1998, 2008; McEwen and Akil, 2020). One key mechanism through which touch may affect health is by modulating allostatic load.

Associated health benefits of touch may depend on relationship context. Romantic partners, as primary attachment figures in adulthood, engage in more frequent touch and such touch may be more powerful than that from other close adults (Shaver and Hazan, 1987; Guerrero, 1997; Sorokowska et al., 2023; Beβler et al., 2020). Simply spending more time in the presence of a partner is associated with lower levels of C-reactive protein (CRP) the following day (Jolink et al., 2023). These benefits extend beyond presence to touch, which has been associated with better long-term psychological well-being (Debrot et al., 2013) and attenuated physiological stress responses, including lower heart rate, blood pressure (Drescher et al., 1980, 1985; Grewen et al., 2003; Lynch et al., 1974; Triscoli et al., 2017), and cortisol (Ditzen et al., 2007). The benefits of touch may not depend on the relationship between the person giving and receiving touch. Platonic touch can also confer benefits (Von Mohr et al., 2017; Heatley Tejada et al., 2020), suggesting that the beneficial effects of touch are not necessarily unique to romantic relationships. Consistent with this, a meta-analysis found no differences in physical or mental health outcomes depending on whether touch was administered by a familiar person (e.g., spouse, friend, or parent) or an unfamiliar person (e.g., experimenter or healthcare professional) (Packheiser et al., 2023). However, this broad distinction between familiar and unfamiliar touch does not establish whether the type of close relationship providing the touch matters. In particular, little is known about whether touch from romantic partners is associated with health differently from touch received from other close adults, such as friends or family members. Understanding whether the source of touch matters may be particularly important because opportunities for interpersonal touch are not equally available to everyone.

Reduced touch is associated with greater stress, anxiety, depression, and loneliness, as well as a greater desire for touch (Beβler et al., 2020; Floyd, 2014; Heatley Tejada et al., 2020; Heidinger and Richter, 2020; Palgi et al., 2020; Stein and Sanfilipo, 1985; Von Mohr et al., 2021). Access to interpersonal touch varies across individuals and contexts, including with age and interpersonal distance preferences (Sorokowska et al., 2021). Thus, if touch from different types of close relationships has differential health associations, differences in people's access to these relationships could have implications for their health and well-being. This may be particularly relevant in later life, when opportunities for touch from romantic partners, friends, and family members may change. Older adults tend to rate touch as more pleasant and describe it in more emotionally expressive terms than younger adults (Guest et al., 2014; Sehlstedt et al., 2016), yet living alone and having smaller social networks may limit opportunities for interpersonal touch among older adults (ONS, 2021; Pedell et al., 2010; Pino et al., 2014; Scharf and de Jong Gierveld, 2008). Thus, touch may be both less accessible and more salient in later life, potentially increasing its relevance for physiological health. Consistent with this possibility, recent evidence suggests that touch may confer stronger systolic blood pressure benefits in older adults (Packheiser et al., 2024a, Packheiser et al., 2024b).

In our previous study (Navyte et al., 2024), we examined associations between touch frequency with romantic partners and other close adults and allostatic load among older adults using data from the National Social Life, Health, and Aging Project (NSHAP). We found that allostatic load in this population did not operate as a single unified system but rather as three distinct physiological systems: metabolic, cardiovascular, and neuroendocrine, with each system capturing physiological burden within its respective domain. Greater touch frequency with romantic partners was associated with more favourable neuroendocrine health, indexed by pulse rate and dehydroepiandrosterone (DHEA) levels, which were assessed concurrently with respondents' reports of touch frequency over the preceding 12 months. These findings provide preliminary evidence that touch within romantic relationships may be associated with variation in neuroendocrine stress-related markers. However, because touch frequency and physiological health were assessed at the same time, the temporal direction of this association could not be established. It therefore remains unclear whether greater touch frequency is associated with subsequent physiological health.

A growing body of research has examined associations between touch and psychological and physiological outcomes over time. Studies using repeated daily assessments have shown that touch is associated with day-to-day variation in affect, mood, stress, and physiological markers. In a 14-day daily diary study, receiving a hug was associated with smaller conflict-related changes in positive and negative affect, both concurrently and in negative affect on the following day (Murphy et al., 2018). Similarly, higher daily embracing was associated with better daily moods over seven days, with greater embracing also associated with higher life satisfaction among single participants (Packheiser et al., 2022), while greater daily hugging was associated with better momentary mood in a later ecological momentary assessment (EMA) study (Packheiser et al., 2024a, Packheiser et al., 2024b). Across two-day EMA studies involving six assessments per day, greater social or affectionate touch was associated with lower burden, anxiety, and stress and higher oxytocin levels (Schneider et al., 2021, 2023).

Other prospective and experimental studies have examined whether touch is associated with subsequent health-related outcomes over periods ranging from the following day to several months. Frequent partner touch has been associated with higher oxytocin and lower blood pressure the following day (Light et al., 2005), while more frequent hugging over 14 days was associated with faster recovery from the common cold (Cohen et al., 2015). Increasing touch over four weeks was associated with higher oxytocin and lower cortisol in both partners and lower blood pressure in men (Holt-Lunstad et al., 2008), while more frequent kissing over six weeks was associated with improved cholesterol levels (Floyd et al., 2009), and massage over 16 weeks was associated with higher dopamine and serotonin, and reduced cortisol and norepinephrine in pregnant women (Field et al., 2004). Reviews of massage research have documented physiological effects following both single and repeated massage sessions (Field, 2016). Increasing cuddling over four weeks has also been shown to improve relational quality among married couples (Van Raalte et al., 2021). However, relatively little research has examined whether everyday touch frequency is associated with changes across multiple domains of physiological health over time. Among older adults, Thomas and Kim (2021) found that frequent physical touch was associated with a lower likelihood of elevated chronic inflammation over time. Nevertheless, evidence linking everyday touch frequency to multiple physiological systems remains limited. It therefore remains unclear whether touch frequency is associated with subsequent metabolic, cardiovascular, and neuroendocrine health, particularly in older adults.

The present study addresses this question separately from our previous work (Navyte et al., 2024), using a different analytic sample from NSHAP and a longitudinal design that permits temporal ordering to be examined. Specifically, we examine whether the frequency of touch with romantic partners and other close adults is associated with metabolic, cardiovascular, and neuroendocrine health assessed five years later. We also examine the reverse temporal ordering, testing whether metabolic, cardiovascular, and neuroendocrine health at the initial assessment is associated with touch frequency five years later. This approach allows us to determine whether associations between touch frequency and physiological health extend prospectively in either direction. Although touch is a multidimensional construct, frequency has been widely used as an indicator of everyday touch exposure and has been consistently associated with physiological outcomes. We hypothesise that (1) more frequent physical touch will be associated with more favourable metabolic, cardiovascular, and neuroendocrine health, and (2) these associations will be stronger for touch with romantic partners than for touch with other close adults.

## 2 Method

### 2.1 Participants

We used data from the National Social Life, Health and Aging Project (NSHAP), a longitudinal survey investigating the multifaceted influences on health and ageing among older adults in the United States. This population-based study collected data from community-dwelling older adults via face-to-face interviews, the collection of biological samples, and the distribution of leave-behind questionnaires among a randomly selected subset of respondents. The survey employed a complex, multistage area probability design with post-stratification and oversampled Black, Hispanic, and older adults aged 75-85 years.

This analysis uses data from Wave 1 and Wave 2 of the NSHAP, conducted from 2005 to 2006 and 2010 to 2011, respectively. Wave 1 included 3005 adults aged 57-85 years (1455 men and 1550 women), with an overall response rate of 75.5%. Wave 2 included 3377 adults aged 62-91 years, comprising respondents from Wave 1, their spouses/partners, and individuals who were sampled but did not participate in Wave 1. Among Wave 2 respondents, 2261 were also respondents in Wave 1 (1085 men and 1176 women). Non-participation in Wave 2 among Wave 1 respondents was attributed to death, relocation, or health issues precluding participation. The overall response rate for Wave 2 was 76.1%. Biomarker collection was conducted at both waves, though limited to a subset of respondents during each wave.

To our knowledge, the NSHAP is the sole nationally representative survey encompassing longitudinal assessments of both non-sexual touch between adults and biomarkers in the USA. Our analytic sample comprises 787 respondents from Wave 2 who also participated in Wave 1 and had complete outcome data across both waves. Of these respondents, 553 (70.3%) reported being in a romantic partnership at Wave 1, while 234 (29.7%) were not partnered. A comparison of the analytic sample and the non-analytic sample (n = 1474) characteristics can be found in Supplementary Table A1.

### 2.2 Touch frequency

Touch frequency was assessed using four questions, two from the Wave 1 questionnaire and two from the Wave 2 questionnaire, which measured touch frequency with romantic partners and other close adults. In Wave 1, respondents were asked “In the last 12 months, how often have you engaged in the following activities: hugging, kissing, caressing, or other close physical contact with your partner?” and “In the last 12 months, how often have you engaged in the following activities: hugging, holding, or other close physical contact with another adult?“. The NSHAP defined “other adults” in Wave 1 as family members, neighbours and friends.

In Wave 2, the wording of these questions was modified. Respondents were asked, “In the last 12 months, how often have you engaged in the following activities? How often have you or your partner shared caring touch, such as a hug, sitting or lying cuddled up, a neck rub or holding hands?” and “Other than your partner, how often have you and a person, such as a friend, grandchild or another adult, shared caring touch, such as a greeting hug, a touch on the arm, or a neck rub? (In the last 12 months, how often have you engaged in the following activities?)”. Each item at each wave was treated as a separate ordinal measure of touch frequency for romantic partners and other close adults at Time 1 and Time 2.

In Wave 1, respondents answered using a 7-point scale ranging from 0 (never) to 6 (several times a week), whereas in Wave 2, the equivalent scale ranged from 0 (never) to 6 (many times a day). To facilitate longitudinal comparisons, response categories were harmonised across waves. Specifically, responses were recoded into four ordinal categories: 1 (never), 2 (once a month), 3 (once a week), and 4 (several times a week or more). In Wave 2, responses indicating “many times a day” were combined with “several times a week” to represent the highest frequency category.

### 2.3 Allostatic load (AL)

Allostatic load was operationalised using three theoretically informed physiological domains: metabolic, cardiovascular, and neuroendocrine health. Each domain was modelled as a latent variable at both Wave 1 and Wave 2 using structural equation modelling (SEM). The metabolic domain was indicated by waist circumference, body mass index (BMI), C-reactive protein (CRP), and Glycosylated haemoglobin (HbA1c); the cardiovascular domain by systolic (sBP) and diastolic blood pressure (dBP); and the neuroendocrine domain by pulse and dehydroepiandrosterone (DHEA).

The biomarker groupings were specified a priori based on allostatic load theory, which distinguishes primary mediators of stress-related activation from downstream metabolic and cardiovascular consequences (Seeman et al., 2001). They were also supported by principal component analysis (PCA) in a partially overlapping sample from the same dataset (Navyte et al., 2024). Although some biomarkers are not specific to a single physiological system, pulse was retained in the neuroendocrine domain because resting pulse reflects autonomic regulation and sympathetic-parasympathetic activity. CRP was retained in the metabolic domain because it represents a downstream component of allostatic load and clustered with the metabolic indicators in the previous PCA (Navyte et al., 2024).

Latent variables were estimated using confirmatory factor analysis (CFA) within the SEM framework, allowing measurement error to be modelled and longitudinal stability to be accounted for. For the cardiovascular and neuroendocrine domains, which each comprised two indicators, factor loadings were constrained to equality across indicators and waves to ensure model identification and longitudinal measurement consistency. Factor loadings for the four metabolic indicators were freely estimated. All indicators loaded significantly and in the expected direction on their respective domains. This CFA approach differs from our previous study (Navyte et al., 2024), in which PCA was used to derive composite allostatic load indices, and was adopted here to permit direct estimation of associations between interpersonal touch and specific physiological domains over time, while retaining conceptual continuity with the earlier PCA-derived groupings.

CRP, HbA1c, and DHEA were log-transformed to address non-normality. All biomarkers were standardised within wave (mean = 0, SD = 1) to facilitate comparability across measures with different units and to ensure consistent interpretation of parameter estimates. Respondents taking antihypertensive or antidiabetic medication and those with CRP values > 10 mg/L were retained to maximise sample retention and reflect physiological variation in the population. Sociodemographic characteristics of respondents taking relevant medications are reported in Supplementary Table A2. The eight biomarkers included at each wave are presented in Table 1.

**Table 1:** Operationalisation of biomarkers used to construct latent allostatic load domains in the NSHAP.

Biomarker	Unit	Latent construct	Transformation	Construct	Method
*C-reactive protein (CRP)*	mg/L	Metabolic	Log-transformed, standardised	Chronic inflammation	A minimum of 250 μL blood was collected from the middle finger (non-dominant hand preferred). CRP was assayed via enzyme-linked immunosorbent assay (McDade et al., 2004), and HbA1c was analysed using the Roche Unimate immunoassay with the Cobas Integra Analyzer
*Glycosylated haemoglobin (HbA1c)*	mmol/mol	Metabolic	Log-transformed, standardised	Glycaemic regulation	
*Body mass index (BMI)*	kg/m^2^	Metabolic	Standardised	Anthropometric functioning	Weight was measured in pounds and height to the nearest half inch
*Waist Circumference*	inches	Metabolic	Standardised		Measured at the narrowest part of the torso above the navel
*Systolic blood pressure (sBP)*	mmHg	Cardiovascular	Standardised	Cardiovascular activity	Mean of two readings taken 1 minute apart using a digital blood pressure monitor
*Diastolic blood pressure (dBP)*	mmHg	Cardiovascular	Standardised		
*Pulse*	beats per minute	Neuroendocrine	Standardised		
*Dehydroepiandrosterone (DHEA)*	μmol/l	Neuroendocrine	Log-transformed, standardised	Adrenal gland steroid	2 mL of saliva collected via chewable sponge; DHEA values averaged across two identical enzyme immunoassays

*Note.* All biomarkers were standardised within wave (mean = 0, SD = 1). Latent-domain classifications reflect empirical biomarker groupings within the allostatic load framework; individual biomarkers may reflect multiple physiological systems.

### 2.4 Covariates

Unless otherwise specified, covariates were from Wave 1 of the NSHAP.

#### 2.4.1 Sexual touch

Sexual touch was categorised into three groups: (1) no sex in the last 12 months, (2) infrequent sexual touch, and (3) frequent sexual touch. These categories were derived from responses to two items.

1. “Had sex in the last year?” (yes/no).
2. Participants who responded affirmatively were then asked: “When you had sex with your partner in the last 12 months, how often did your activities include kissing, hugging, caressing, or other ways of sexual touching?” (1 (never) to 5 (always)).

Respondents reporting sexual touch “always” (5) were classified as having frequent sexual touch, whereas those reporting frequencies of 1-4 were classified as having infrequent sexual touch. The latter categories were collapsed because of small cell sizes within individual response categories.

#### 2.4.2 Alcohol intake

Alcohol consumption was assessed using three survey items: (1) “Have you ever drunk alcohol?” (yes/no), (2) “Do you drink alcohol?” (yes/no), and (3) “How many days per week do you drink?” (0 (none or < 1/wk) −7 (every day)). Participants were classified into three groups: (1) regular drinkers, reporting alcohol intake on at least one day per week, (2) non-regular drinkers, reporting alcohol intake on less than one day per week, and (3) never drank alcohol. The threshold of one day per week was chosen to capture participants with recurring, habitual alcohol use while maintaining sufficient group sizes for analysis.

#### 2.4.3 Household composition

Number of co-residents was categorised into three groups: (1) lives alone, (2) lives with one other person, and (3) lives with two or more persons.

#### 2.4.4 Romantic partner

The romantic partner variable was binary. One group comprised individuals who were in a romantic relationship at the time of Wave 1 data collection, including those who were married, cohabiting, or involved in a romantic, intimate, or sexual relationship. The other group consisted of individuals who were not in a romantic relationship at the time of the Wave 1 survey, including those who were separated, divorced, widowed, never married, and not involved in a romantic, intimate, or sexual relationship.

#### 2.4.5 Sociodemographic characteristics

All sociodemographic variables used were from Wave 1, however total household assets were only measured at Wave 2. Sex and ethnicity were measured as binary variables: men and women; White and non-White (including Black and Hispanic respondents). Age was categorised into three groups: (1) 57-64, (2) 65-74, and (3) 75-85 years in descriptive analyses.

Socioeconomic position (SEP) combined total household assets (property, cars, businesses, financial assets etc.) and educational attainment. Educational attainment was a categorical variable with four groups: (1) less than high school, (2) high school degree or equivalent, (3) some college or associate degree, and (4) bachelor's degree or higher. Total household assets were also categorised into four groups: (1) $0-49,999, (2) $50,000-99,999, (3) $100,000-499,999, and (4) $500,000 or more.

#### 2.4.6 Relationship happiness

Relationship happiness was assessed at Wave 1 using a single item asking respondents, “Taking all things together, how would you describe your marriage/relationship with [partner]?” Responses were provided on a 7-point scale ranging from 1 (very unhappy) to 7 (very happy), with higher scores indicating greater relationship happiness. This variable was included as an additional covariate in sensitivity analyses examining whether associations between romantic partner touch and subsequent health were robust to adjustment for relationship happiness.

### 2.5 Statistical analyses

Structural equation modelling (SEM) was used to examine longitudinal associations between romantic partner touch frequency, touch frequency with other close adults, and three latent health outcomes: metabolic, cardiovascular, and neuroendocrine health, across two waves of the NSHAP. Cross-lagged path models were specified to assess bidirectional associations between touch variables and health outcomes over time.

Each health outcome was modelled as a latent variable at both Wave 1 and 2 of the NSHAP. Latent variables were specified using observed biomarker indicators corresponding to metabolic, cardiovascular, and neuroendocrine domains. Autoregressive paths were included for all latent health variables and touch measures, and cross-lagged paths were specified to test longitudinal associations between touch frequency and health outcomes across waves. The cross-lagged path models specified that each latent outcome variable at Wave 2 was regressed on its corresponding latent variable at Wave 1 and touch frequency (either romantic partner or other close adult) at Wave 1. Additionally, touch frequency at Wave 2 was regressed on touch frequency at Wave 1 and the latent variables at Wave 1.

Sociodemographic characteristics (age, sex, ethnicity, educational attainment, and household assets) were included in the minimally adjusted Model 1 (base model). Further adjustments were made in additional models to include household composition, romantic partner status (only for touch frequency with other close adults’ models), sexual touch, and alcohol intake. This resulted in a total of five models for each combination of health outcome (metabolic, cardiovascular, and neuroendocrine health) and touch frequency source (romantic partner and other close adults).

The models were fitted using the ‘sem’ function from the ‘lavaan’ package in R, employing full information maximum likelihood (FIML) to account for missing data as recommended for structural equation modelling for NSHAP data (Hawkley et al., 2014). Romantic partner touch was only measured among participants with a current partner, whereas touch with other close adults was assessed in all respondents; unpartnered participants were retained in the analytic sample and handled via FIML, resulting in a shared analytic sample frame (N = 787) across models. The NSHAP does not yet have a true panel weight for longitudinal analyses.

#### 2.5.1 Supplementary and sensitivity analyses

Several supplementary and sensitivity analyses were conducted to assess the robustness and interpretation of the primary findings. First, to examine which individual biomarkers contributed to associations observed for the latent health domains, the primary cross-lagged models were re-estimated separately for each biomarker comprising the metabolic, cardiovascular, and neuroendocrine constructs. These analyses examined associations between Wave 1 romantic partner touch and each individual biomarker at Wave 2, while retaining the corresponding autoregressive and covariate paths from the primary models.

Second, sensitivity analyses were conducted to assess the potential influence of medication use and elevated inflammation on the findings. The primary models were re-estimated with additional adjustment for antihypertensive and antidiabetic medication use. Models were also re-estimated after excluding respondents with CRP values > 10 mg/L, a commonly used threshold indicating potentially acute inflammation. Third, to examine whether associations between romantic partner touch and subsequent health were attributable to differences in relationship happiness, sensitivity analyses additionally adjusted for relationship happiness assessed at Wave 1. The same cross-lagged models were re-estimated for metabolic, cardiovascular, and neuroendocrine health, with relationship happiness included as a covariate predicting both Wave 2 health and Wave 2 romantic partner touch. Estimates were compared with those from the corresponding primary models. Full results of these supplementary and sensitivity analyses are reported in Supplementary Tables A6–A.7.

## 3 Results

### 3.1 Participant characteristics

Table 2 presents the distribution of romantic partner and other adult touch frequency across key sociodemographic characteristics and covariates in the final Wave 1 sample (n = 787) from NSHAP. Corresponding Wave 2 characteristics and additional covariates are reported in Supplementary Table A3. Wave 1 sociodemographic characteristics and covariates were included in all adjusted analyses. Bivariate Spearman correlations among all observed variables included in the structural equation models, including touch measures, biomarkers, and covariates, are reported in Supplementary Table A4.

**Table 2:** Descriptive statistics of touch frequency (from romantic partners and other adults), sociodemographic variables, and covariates in the final sample (n = 787) from NSHAP Wave 1 (2005 to 2006).

Demographic Characteristic	Partner Touch Frequency (%)				Touch with Others Frequency (%)			
	Never	Monthly	Weekly	>Weekly	Never	Monthly	Weekly	>Weekly
**Women**^a^	50.00	38.98	42.55	35.54	37.17	42.60	60.24	60.76
**White**^b^	25.00	62.07	75.56	85.18	70.59	82.03	77.78	74.03
**Age (y)**								
57-64	37.50	38.98	42.55	43.38	34.55	40.81	35.54	36.71
65-74	37.50	37.29	44.68	38.48	41.88	35.87	37.95	45.57
75-85	25.00	23.73	12.77	18.14	23.56	23.32	26.51	17.72
**Educational Attainment**								
<High School	37.50	23.73	10.64	12.99	18.85	14.80	16.27	18.99
High School Degree or Equivalent	12.50	32.20	29.79	24.75	30.89	26.46	18.07	21.52
Some College or Associate Degree	37.50	32.20	25.53	32.35	32.46	29.15	36.14	33.54
Bachelor's Degree or Higher	12.50	11.86	34.04	29.90	17.80	29.60	29.52	25.95
**Total Household Assets ($)**^c^								
0-49,999	50.00	10.87	11.43	9.22	19.26	15.03	8.33	22.43
50,000–99,999	16.67	8.70	5.71	7.45	10.37	8.67	12.04	13.08
100,000–499,999	16.67	54.35	42.86	37.94	36.30	45.09	42.59	31.78
500k or higher	16.67	26.09	40.00	45.39	34.07	31.21	37.04	32.71

*Note.* Percentages represent the proportion of respondents within each touch-frequency category who belong to each demographic group at Wave 1. For example, among respondents who reported “Never” engaging in partner touch, 50.00% were women and 50.00% were men. Thus, percentages within each touch-frequency category sum to 100% across the categories of the corresponding demographic characteristic. N's for each touch group are not reported as FIML was used.

a Women compared to men.

b White respondents compared to Black and Hispanic respondents.

c Only measured at Wave 2.

### 3.2 Cross-lagged path models

Table 3 presents the focal cross-lagged path associations with Wave 2 latent health domains (metabolic, cardiovascular, neuroendocrine) and Wave 2 touch frequency (romantic partners and other close adults) from Wave 1 predictors. Five nested models were estimated for each predictor.

Model 1 **(base model):** Adjusted for sociodemographic characteristics (age, sex, ethnicity, educational attainment, and total household assets).
Model 2: Model 1 + household composition.
Model 3: Model 2 + romantic partner status.
Model 4: Model 3 + frequency of sexual touch.
Model 5 **(full model):** Model 4 + alcohol intake.

**Table 3:** *Cross-lagged path models examining bidirectional longitudinal associations between touch frequency (with romantic partners and other close adults) at Wave 1 (2005 to 2006) and latent health outcomes (metabolic, cardiovascular, and neuroendocrine) at Wave 2 (2010 to 2011)*.

Predictors	Wave 2 Metabolic Health		Wave 2 Cardiovascular Health		Wave 2 Neuroendocrine Health		Wave 2 Romantic Partner Touch Frequency		Wave 2 Other Close Adult Touch Frequency	
	*B*	95% CI	*B*	95% CI	*B*	95% CI	*B*	95% CI	*B*	95% CI
Wave 1 Metabolic Health										
Model 1 (base model)	1.01**	0.96-1.07					−0.01	−0.10-0.09	0.07	−0.02-0.16
Model 2: Model 1 + household composition	1.01**	0.96-1.07					−0.01	−0.11-0.09	0.07	−0.02-0.16
Model 3: Model 2 + romantic partner	1.01**	0.95-1.06					−0.01	−0.11-0.09	0.07	−0.02-0.15
Model 4: Model 3 + sexual touch	1.01**	0.95-1.06					−0.01	−0.11-0.09	0.07	−0.02-0.16
Model 5: Model 4 + alcohol intake	1.00**	0.95-1.06					−0.00	−0.10-0.09	0.07	−0.02-0.16
Wave 1 Cardiovascular Health										
Model 1 (base model)			0.60**	0.51-0.69			0.003	−0.11-0.11	−0.02	−0.13-0.08
Model 2: Model 1 + household composition			0.60**	0.51-0.69			0.01	−0.11-0.12	−0.02	−0.13-0.08
Model 3: Model 2 + romantic partner			0.60**	0.51-0.69			0.01	−0.11-0.12	−0.02	−0.13-0.08
Model 4: Model 3 + sexual touch			0.60**	0.51-0.69			0.01	−0.10-0.12	−0.02	−0.13-0.08
Model 5: Model 4 + alcohol intake			0.60**	0.51-0.69			0.01	−0.10-0.12	−0.02	−0.13-0.09
Wave 1 Neuroendocrine Health										
Model 1 (base model)					1.57**	0.55-2.58	0.15	−0.42-0.73	0.01	−0.51-0.54
Model 2: Model 1 + household composition					1.57**	0.56-2.59	0.15	−0.42-0.72	0.01	−0.51-0.54
Model 3: Model 2 + romantic partner					1.56**	0.55-2.56	0.15	−0.43-0.72	0.00	−0.52-0.52
Model 4: Model 3 + sexual touch					1.56**	0.55-2.57	0.15	−0.42-0.72	−0.01	−0.53-0.52
Model 5: Model 4 + alcohol intake					1.57**	0.55-2.58	0.12	−0.46-0.69	0.01	−0.51-0.53
Wave 1 Romantic Partner Touch Frequency										
Model 1 (base model)	−0.07**	−0.13 to −0.02	−0.11*	−0.21 to −0.02	0.03	−0.05-0.10	0.65**	0.55-0.75		
Model 2: Model 1 + household composition	−0.07**	−0.13 to −0.02	−0.11*	−0.21 to −0.02	0.02	−0.05-0.10	0.65**	0.55-0.75		
Model 3: Model 2 + romantic partner	−0.07**	−0.13 to −0.02	−0.11*	−0.21 to −0.02	0.02	−0.05-0.10	0.65**	0.55-0.75		
Model 4: Model 3 + sexual touch	−0.08**	−0.13 to −0.02	−0.14**	−0.23 to −0.04	0.04	−0.04-0.12	0.63**	0.52-0.74		
Model 5: Model 4 + alcohol intake	−0.08**	−0.13 to −0.02	−0.14**	−0.23 to −0.04	0.04	−0.04-0.12	0.63**	0.52-0.74		
Wave 1 Other Close Adult Touch Frequency										
Model 1 (base model)	−0.00	−0.03-0.03	0.01	−0.05-0.06	0.03	−0.02-0.07			0.23**	0.17-0.30
Model 2: Model 1 + household composition	−0.00	−0.03-0.03	0.01	−0.05-0.06	0.02	−0.02-0.07			0.24**	0.17-0.30
Model 3: Model 2 + romantic partner	−0.00	−0.03-0.03	0.01	−0.05-0.06	0.02	−0.02-0.07			0.24**	0.17-0.30
Model 4: Model 3 + sexual touch	−0.00	−0.03-0.03	0.00	−0.05-0.06	0.02	−0.02-0.07			0.23**	0.16-0.30
Model 5: Model 4 + alcohol intake	−0.00	−0.03-0.03	0.01	−0.05-0.06	0.03	−0.02-0.07			0.23**	0.17-0.30

*Note.* All coefficients are standardised, and 95% confidence intervals are reported. Model 1 (base model) is adjusted for sociodemographic characteristics (age, sex, ethnicity, educational attainment, and total household assets). Negative *B* values indicate better health outcomes. Positive *B* values indicate worse health outcomes, suggesting more load on the respective system. *p < .05. **p < .01. Full parameter estimates for all predictors and covariates included in the final models are provided in Supplementary Table A5.

**Fig. 1:**
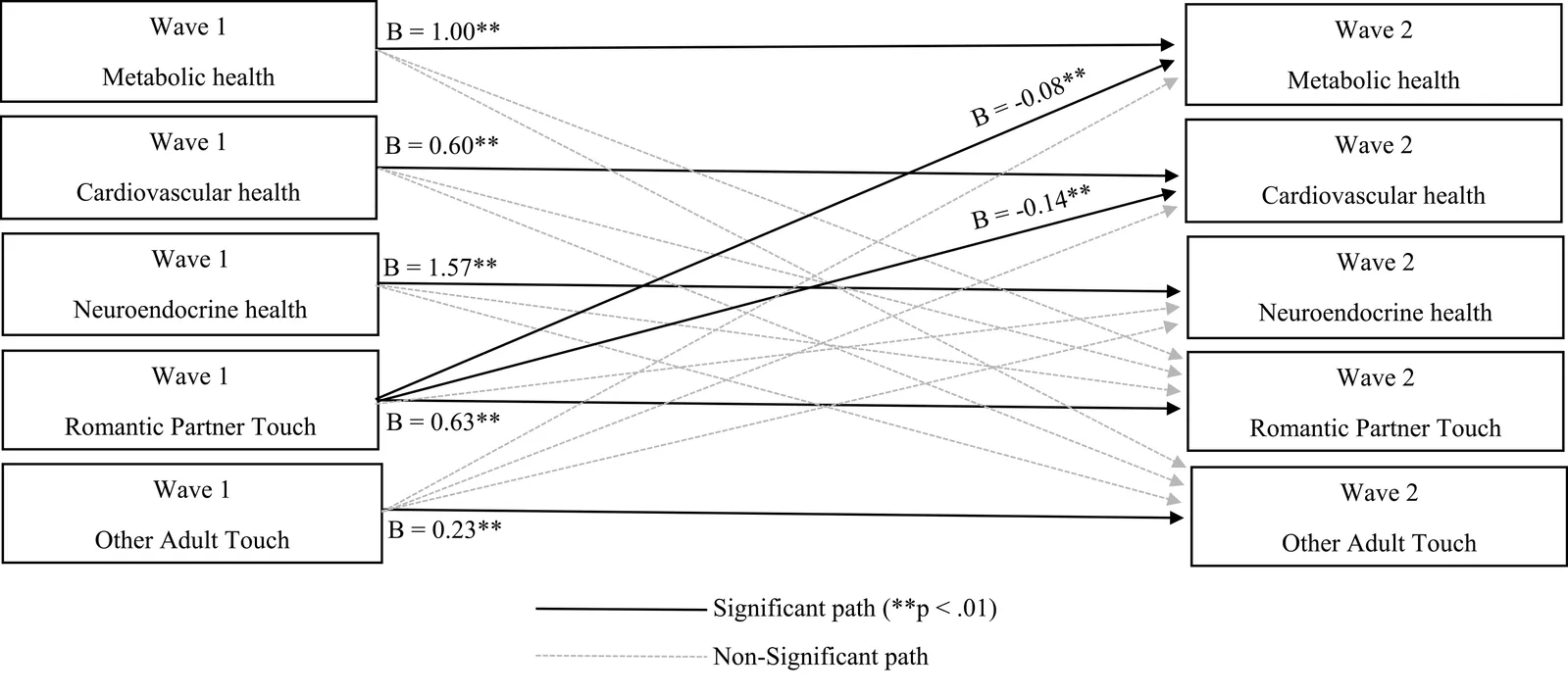
Cross-lagged associations between Wave 1 and 2 metabolic, cardiovascular, and neuroendocrine health variables, and touch frequency variables.

Fig. 1 illustrates the cross-lagged associations between Wave 1 and wave 2 latent health domains (metabolic, cardiovascular, and neuroendocrine) and touch frequency with romantic partners and other close adults, corresponding to the results reported in Table 3.

#### 3.2.1 Metabolic health

Wave 1 metabolic health was associated with Wave 2 metabolic health across all models (Model 5: *b* = 1.00, *p* = <0.001), indicating temporal stability in metabolic physiological load over the 5-year period. However, Wave 1 metabolic health was not associated with Wave 2 touch frequency in any model (Model 5: romantic partner *b* = −0.00, *p* = .931; other adults *b* = 0.07, *p* = .132).

**Fig. 2:**
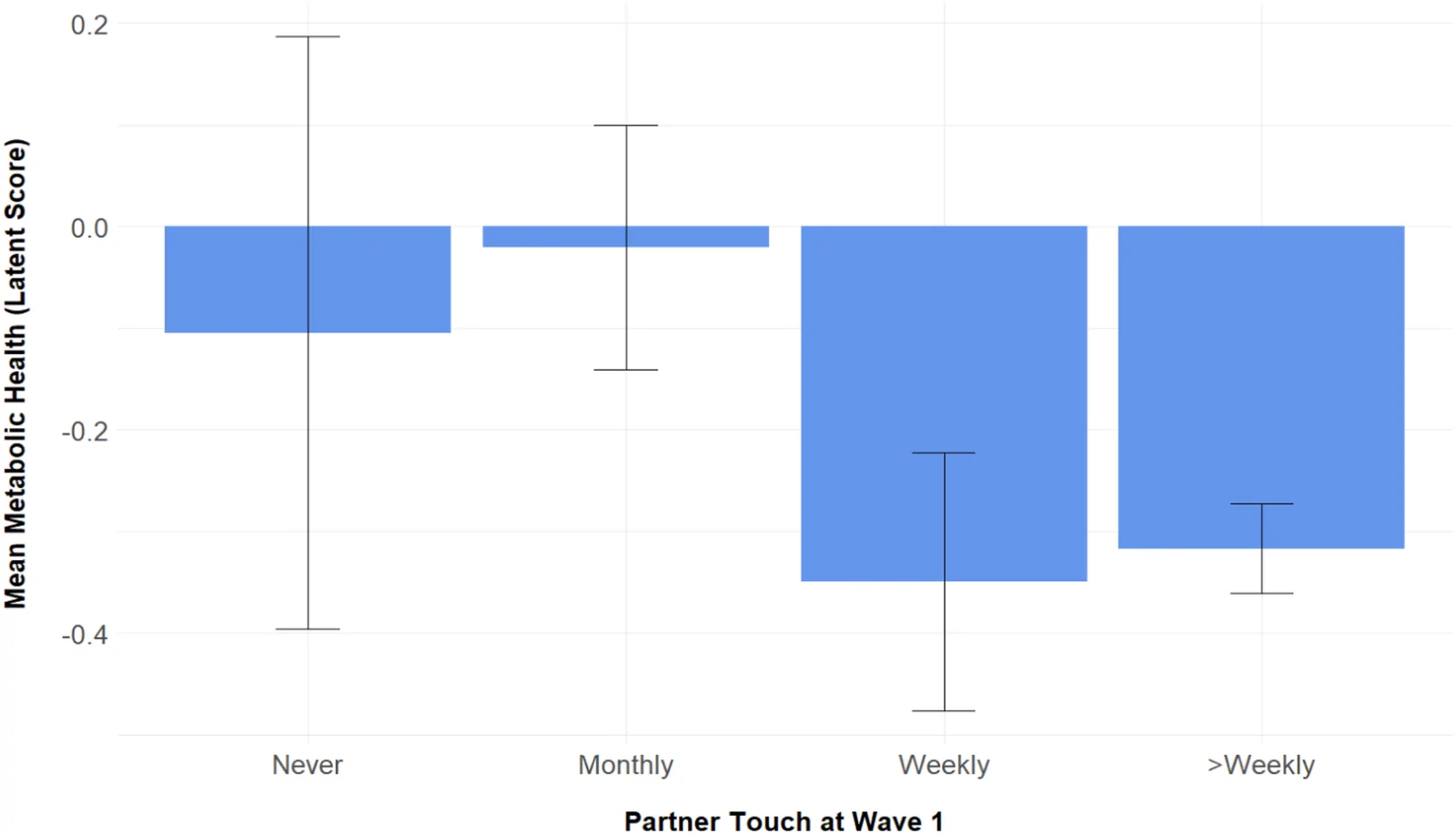
*Metabolic health at Wave 2 (2010 to 2011) by increasing romantic partner touch frequency at Wave 1((2005 to 2006) of the NSHAP* *Note. Metabolic health* at Wave 2 was derived from standardised values of waist circumference, CRP, HbA1c, and BMI. The Scale represents deviations from the sample mean metabolic health at Wave 2. Negative values indicate better metabolic health, with metabolic indicators below the average. Conversely, positive values indicate worse metabolic health compared to the sample mean. This Figure presents observed group means, with error bars representing the standard error of the mean (SEM).

Higher romantic partner touch frequency at Wave 1 was associated with lower metabolic physiological load at Wave 2 (Model 5: *b* = −0.08, *p* = .006), indicating more favourable metabolic health (see Fig. 2). This relationship was stable across Models 1-5, indicating robustness to covariate and lagged metabolic health adjustment (see Table 3). Each unit increase in romantic partner touch frequency (e.g., from ‘Monthly’ to ‘Weekly’) was associated with a 0.08 standard deviation reduction in metabolic dysregulation at Wave 2. More frequent touch with other close adults at Wave 1 was not associated with metabolic health at Wave 2 (Model 5: *b* = −0.00, *p* = .993).

#### 3.2.2 Cardiovascular health

Wave 1 cardiovascular health was associated with cardiovascular health at Wave 2 across all models (Model 5: *b* = 0.60, *p* = <0.001), indicating temporal stability in cardiovascular physiological load over time. *Cardiovascular health* at Wave 1 was not associated with touch frequency at Wave 2 (Model 5: romantic partner *b* = −0.01, *p* = .861; other adults *b* = −0.02, *p* = .727).

**Fig. 3:**
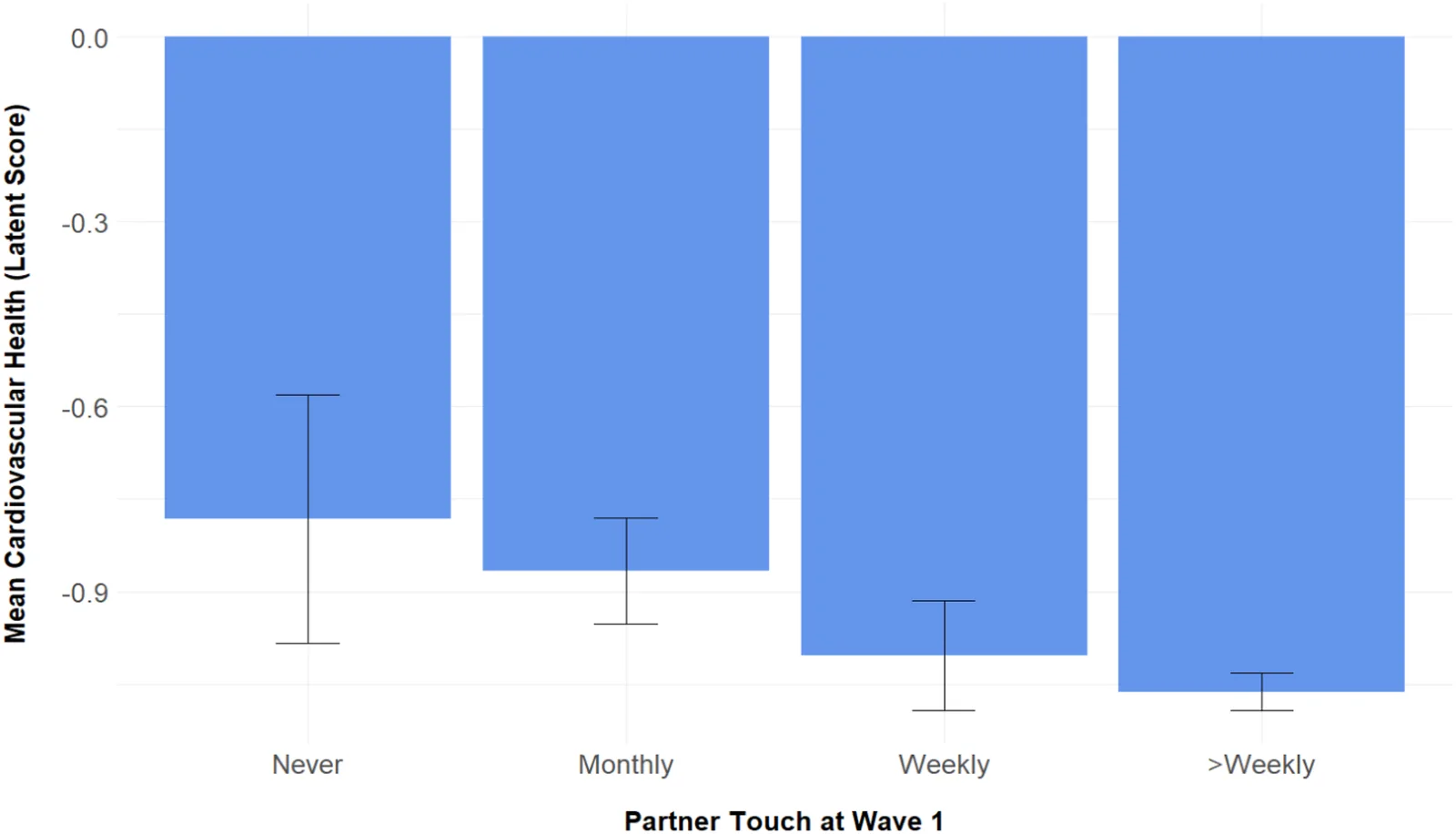
*Cardiovascular health at Wave 2 (2010 to 2011) by increasing romantic partner touch frequency at Wave 1 ((2005 to 2006) of the NSHAP* *Note. Cardiovascular health* at Wave 2 was derived from standardised values of SBP and DBP. The scale represents deviations from the sample mean cardiovascular health at Wave 2. Negative values indicate better cardiovascular health, with cardiovascular health indicators below the average, whereas positive values indicate worse cardiovascular health. The Figure presents observed group means, with error bars representing the SEM.

Higher romantic partner touch frequency at Wave 1 was associated with lower cardiovascular load at Wave 2 (Model 5: *b* = −0.14, *p* = .005), indicating more favourable cardiovascular health (see Fig. 3). Each unit increase in romantic partner touch frequency (e.g., from ‘Monthly’ to ‘Weekly’) corresponded to a 0.14 standard deviation reduction in cardiovascular physiological load at Wave 2. More frequent touch with other close adults at Wave 1 was not associated with cardiovascular health at Wave 2 (Model 5: *b* = 0.01, *p* = .851).

### 3.3 Neuroendocrine health

Wave 1 neuroendocrine health was associated with Wave 2 neuroendocrine health in all models (Model 5: *b* = 1.57, *p* = .002), indicating temporal stability. Touch frequency, either romantic partner or other adults was not associated with Wave 2 neuroendocrine health (Model 5: romantic partner *b* = 0.04, *p* = .327; other adults: *b* = 0.03, *p* = .280), and Wave 1 neuroendocrine health was not associated with Wave 2 touch frequency at Wave 2 (Model 5: romantic partner *b* = 0.12, *p* = .690; other adults *b* = 0.01, *p* = .975).

### 3.4 Supplementary and sensitivity analyses

To confirm robustness, individual biomarkers were examined with partner touch frequency (see Supplementary Table A6). The association between romantic partner touch and metabolic health was most strongly reflected in BMI (*b* = −0.12, *p* = .001), with smaller and non-significant contributions from CRP (*b* = −0.04, *p* = .362), HbA1c (*b* = −0.06, *p* = .156), and waist circumference (*b* = −0.05, *p* = .106). *Cardiovascular health* relationships were primarily driven by sBP (*b* = −0.14, *p* = .011) and dBP (*b* = −0.13, *p* = .013). Neuroendocrine indicators remained non-significant (DHEA: *b* = 0.03, *p* = .629; Pulse: *b* = 0.05, *p* = .340). Models adjusting for medication use and excluding respondents with CRP >10 mg/L showed a comparable pattern of associations, with no material changes in the direction, magnitude, or significance of the main effects.

Results were also robust to additional adjustment for relationship happiness. The associations between romantic partner touch and subsequent metabolic and cardiovascular health were not attenuated following adjustment, while there remained no evidence of an association between romantic partner touch and subsequent neuroendocrine health. Full results of these sensitivity analyses are presented in Supplementary Table A7.

## 4 Discussion

This study investigated whether the frequency of touch from romantic partners, compared with other close adults, was associated with long-term physiological health in older adults. More frequent romantic partner touch was associated with more favourable metabolic and cardiovascular health five years later, and these associations remained robust after adjustment for sociodemographic characteristics and other covariates. In contrast, touch from other close adults was not associated with subsequent health outcomes. Although prior cross-sectional evidence has linked romantic partner touch with neuroendocrine health (Navyte et al., 2024), neither romantic partner nor other close adult touch was associated with neuroendocrine health over time. One possible interpretation is that associations between touch and physiological health may differ according to the timescale over which they are assessed, with neuroendocrine responses potentially being more proximal to experiences of touch, while metabolic and cardiovascular indicators may reflect more cumulative physiological processes. Additionally, baseline health status was not associated with subsequent touch frequency, suggesting that healthier individuals were not simply more likely to engage in frequent touch, and reducing the likelihood that the observed associations are explained by reverse temporal ordering or pre-existing health differences.

Our findings underscore the salience of social support in older adulthood, a life stage often associated with heightened risk of loneliness and social isolation, both of which are consistently linked with poorer health outcomes (ONS, 2021; Pedell et al., 2010; Pino et al., 2014; Scharf and de Jong Gierveld, 2008). Interpersonal touch, as a direct and embodied form of support, has been associated with reduced psychological distress, attenuated physiological stress responses, and lower perceived social threat, while limited access to touch has been associated with greater loneliness (Coan et al., 2006; Ditzen et al., 2007; von Mohr et al., 2017; Heatley Tejada et al., 2020). Extending this literature, largely based on younger samples, the present findings suggest that, in older adults, frequent touch within romantic relationships may index a form of support that is both consistently available and physiologically relevant for metabolic and cardiovascular systems. The absence of comparable associations for touch from other close adults further indicates that social support through touch may not be interchangeable across relationships, and that intimate partnerships may provide more powerful forms of support, highlighting the importance of considering not only the availability of social ties in later life, but also the specific modalities through which support is experienced.

These findings are broadly consistent with prior research using the NSHAP cohort, while also highlighting important differences in the specific physiological pathways involved. For example, Lee and Cichy (2020) reported that higher levels of touch strengthened the association between positive relationship quality and lower systolic blood pressure and pulse in cross-sectional analyses, with no association observed at low touch frequency. Similarly, Thomas and Kim (2021) found that more frequent touch was associated with lower inflammation, indexed by CRP alone, five years later. While we also observed an association between touch and metabolic health, the pattern of results differed in that this association appeared to be primarily driven by BMI rather than CRP. Several factors may account for this divergence. Methodologically, our analyses were restricted to respondents with complete data across all eight allostatic load biomarkers at both waves (N = 787), resulting in a smaller analytic sample, whereas Thomas and Kim (2021) analysed a larger sample (N = 1124) by focusing exclusively on CRP derived from dried blood spot samples. In addition, we modelled touch from romantic partners and other close adults separately, instead of combining these exposures. Our outcome captured a broader metabolic construct, incorporating CRP alongside BMI, waist circumference, and HbA1c, which represent related but distinct physiological processes. These findings suggest that while touch is consistently associated with metabolic health, the specific biological pathways through which these associations are expressed may differ depending on how both exposures and outcomes are operationalised.

The observed associations may be understood within an allostatic framework, in which metabolic, cardiovascular, and neuroendocrine biomarkers index the cumulative physiological consequences of repeated adaptation to environmental and psychosocial demands over time (Sterling and Eyer, 1988; Seeman et al., 2001). Allostatic load reflects multisystem dysregulation that accrues over time in response to chronic or repeated stress exposure, and is commonly operationalised using primary (e.g. stress hormones) and secondary (e.g., raised blood pressure) physiological indicators. Elevated allostatic load contributes to cardiovascular disease (Seeman et al., 2001), cognitive and physical functioning decline (Karlamangla et al., 2002), and all-cause mortality (Goldman et al., 2006). While experimental studies suggest that touch can attenuate acute neuroendocrine responses and enhance social regulation (Ditzen et al., 2007; Navyte et al., 2024), the present findings speak specifically to longer-term differences in secondary allostatic load markers. The present measure captures habitual touch frequency over the preceding 12 months rather than acute responses to individual touch episodes. Thus, associations between habitual touch and long-term physiological health may differ from the immediate physiological responses observed in experimental studies. Stress buffering is one plausible pathway, whereby repeated regulation of stress responses may contribute to cumulative differences in metabolic and cardiovascular health over time.

A key feature of the present findings is the specificity of the observed associations to romantic partner touch, with no comparable associations observed for touch from other close adults. This pattern is particularly noteworthy given evidence that the benefits of touch may extend across different interpersonal contexts. For example, a meta-analysis found no differences in physical or mental health outcomes according to whether touch was administered by a familiar person (e.g., spouse, friend, or parent) or an unfamiliar person (e.g., experimenter or healthcare professional) (Packheiser et al., 2023). Our findings suggest that this broad distinction between familiar and unfamiliar touch may not capture the full relevance of relational context. Although touch may confer benefits across interpersonal contexts, the specific nature of a close relationship may still shape its association with physiological health. Consistent with this possibility, previous research has linked touch within romantic relationships to more favourable cardiovascular and metabolic profiles. For example, partner touch has been associated with lower blood pressure (Holt-Lunstad et al., 2008; Light et al., 2005), while more frequent passionate kissing over six weeks has been linked to reductions in total serum cholesterol (Floyd et al., 2009), and greater trait affection to more favourable vascular and metabolic markers, including blood pressure and HbA1c (Floyd et al., 2007). Although the present study did not directly compare romantic and non-romantic touch within the same statistical model, the absence of associations for touch from other close adults suggests that the health relevance of touch may depend, at least in part, on its relational context.

One possible explanation is that romantic relationships typically involve greater intimacy, emotional salience, and frequency of contact than other close relationships, potentially strengthening the physiological relevance of touch. More broadly, romantic partners may provide a distinctive source of social safety and stress regulation. For instance, having a romantic partner in close proximity has been associated with reduced threat-related amygdala activity over time compared with proximity to a friend (Morriss et al., 2019), and relationship quality has been found to account for up to 16.8% of the stress-alleviating effects of partner touch (Liu et al., 2021). Likewise, an attentive romantic partner can enhance feelings of security during challenging tasks, compared to having an inattentive partner (Kane et al., 2012), while handholding from a romantic partner has been shown to attenuate pain in ways not observed when touch was provided by friends or strangers (Floyd et al., 2018). Together, these findings suggest that the meaning and function of touch may differ according to the relationship in which it occurs, with romantic partner touch potentially carrying particular emotional and regulatory significance. Extending our previous cross-sectional findings (Navyte et al., 2024), the present study suggests that these relationship-specific associations may persist over a five-year period. These findings highlight the importance of considering relational context when examining the potential long-term health correlates of interpersonal touch.

Although this study extends the literature in several important ways, several limitations warrant consideration. First, residual confounding cannot be excluded. Although we adjusted for key sociodemographic and health-related covariates, factors such as physical activity (Erlichman et al., 2002), and individual perceptions of touch may influence health outcomes and were not fully captured. In addition, NSHAP combines all non-romantic close adults into a single category, potentially obscuring variation in the type, context, and frequency of touch across relationships. Cultural norms may further shape the frequency and acceptability of non-romantic touch across cultures, generations, and social groups (Dibiase and Gunnoe, 2004). As NSHAP comprises a US sample, these patterns may not generalise to populations with different norms surrounding interpersonal touch. Future studies should distinguish between relationship types and incorporate culturally diverse samples and measures of attitudes towards touch.

Second, measurement limitations may have introduced additional variability. Touch frequency was assessed using single self-report items, with different wording and response scales across waves, limiting construct precision and increasing susceptibility to recall and social desirability biases. The 12-month retrospective assessment may also provide an imperfect representation of everyday touch. Moreover, the romantic partner measure may conflate affiliative and potentially sexual touch (Sedgwick, 2012). Future research would benefit from psychometrically validated measures combined with high-frequency ecological momentary assessment to capture the frequency, context, and nature or interpersonal touch more precisely. Third, the analytic sample was relatively modest (n = 787), limiting power to detect heterogeneity within the older adult population. We retained respondents taking antihypertensive or antidiabetic medication (n = 268) and those with CRP >10 mg/L (n = 66) to preserve statistical power; excluding these respondents would have reduced the sample to n = 453. Supplementary analyses indicated that adjusting for medication use and excluding respondents with elevated CRP did not materially alter the pattern of findings, although their inclusion may nevertheless contribute additional variability. Finally, the cross-lagged design provides evidence of temporal ordering but cannot establish causality. Residual confounding and other forms of bias may account for observed associations, and stronger causal inference will require designs with more comprehensive confounding control, repeated assessments, or experimental manipulation.

From a public health perspective, these findings suggest that touch within romantic partnerships may represent a low-cost, non-invasive aspect of social connection relevant to healthy ageing. Although the observational design precludes causal conclusions, interventions that promote safe and consensual touch within relationships may warrant consideration as one potential avenue for supporting psychological wellbeing and stress regulation, with potential implications for metabolic and cardiovascular health. Clinically, these findings highlight interpersonal touch as a potentially meaningful dimension of social relationships alongside more established forms of social support. At the population level, incorporating measures of interpersonal touch into assessments of social relationships may provide a more complete understanding of how relational experiences contribute to health. Future research should examine these associations in more diverse populations and across the life course, while using longitudinal and experimental approaches to determine whether, for whom, and under what circumstances interpersonal touch may contribute to long-term health.

## CRediT authorship contribution statement

**Gabriele Navyte:** Conceptualization, Data curation, Formal analysis, Investigation, Methodology, Visualization, Writing – original draft, Writing – review & editing. **Helge Gillmeister:** Supervision, Validation, Writing – review & editing. **Meena Kumari:** Conceptualization, Formal analysis, Methodology, Supervision, Validation, Writing – review & editing.

## Ethics declaration

Written informed consent to take part in the study and to publish the article has been obtained from all participants or their legal representatives. The privacy rights of participants have been observed.

This study was performed in compliance with relevant laws, regulatory frameworks and guidelines where the research took place. Ethics committee approval was not required under relevant laws and institutional guidelines. This study involved secondary analysis of de-identified data from the National Social Life, Health, and Aging Project (NSHAP), an established longitudinal study of older adults. No new data were collected from human participants for the present study; therefore, ethics committee approval was not required for this secondary analysis in accordance with applicable institutional and regulatory requirements.

## Funding

This work was supported by the ESRC-BBSRC Soc-B Centre for Doctoral Training (10.13039/501100018726ES/T00200X/1, project reference 2442358), who funded GN’s PhD.

## Declaration of competing interest

The authors declare the following financial interests/personal relationships which may be considered as potential competing interests: Gabriele Navyte reports financial support was provided by ESRC-BBSRC Soc-B Centre for Doctoral Training. If there are other authors, they declare that they have no known competing financial interests or personal relationships that could have appeared to influence the work reported in this paper.

## References

[bib2] Berkman L.F., Glass T., Brissette I., Seeman T.E. (2000). From social integration to health: Durkheim in the new millennium. Soc. Sci. Med..

[bib1] Beßler R., Bendas J., Sailer U., Croy I. (2020). The “longing for interpersonal touch picture questionnaire”: development of a new measurement for touch perception. Int. J. Psychol..

[bib3] Bowlby J. (1982). Attachment and loss: retrospect and prospect. Am. J. Orthopsychiatry.

[bib4] Carter C.S. (1998). Neuroendocrine perspectives on social attachment and love. Psychoneuroendocrinology.

[bib5] Che X., Cash R., Chung S., Fitzgerald P.B., Fitzgibbon B.M. (2018). Investigating the influence of social support on experimental pain and related physiological arousal: a systematic review and meta-analysis. Neurosci. Biobehav. Rev..

[bib6] Coan J.A., Schaefer H.S., Davidson R.J. (2006). Lending a hand: social regulation of the neural response to threat. Psychol. Sci..

[bib7] Cohen S. (1988). Psychosocial models of the role of social support in the etiology of physical disease. Health Psychol..

[bib8] Cohen S., Janicki-Deverts D., Turner R.B., Doyle W.J. (2015). Does hugging provide stress-buffering social support? A study of susceptibility to upper respiratory infection and illness. Psychol. Sci..

[bib9] Coleman D., Iso-Ahola S.E. (1993). Leisure and health: the role of social support and self-determination. J. Leisure Res..

[bib10] Debrot A., Schoebi D., Perrez M., Horn A.B. (2013). Touch as an interpersonal emotion regulation process in couples' daily lives: the mediating role of psychological intimacy. Pers. Soc. Psychol. Bull..

[bib11] Dibiase R., Gunnoe J. (2004). Gender and culture differences in touching behavior. J. Soc. Psychol..

[bib12] Ditzen B., Neumann I.D., Bodenmann G., von Dawans B., Turner R.A., Ehlert U., Heinrichs M. (2007). Effects of different kinds of couple interaction on cortisol and heart rate responses to stress in women. Psychoneuroendocrinology.

[bib13] Dreisoerner A., Junker N.M., Schlotz W., Heimrich J., Bloemeke S., Ditzen B., van Dick R. (2021). Self-soothing touch and being hugged reduce cortisol responses to stress: a randomized controlled trial on stress, physical touch, and social identity. Comprehensive Psychoneuroendocrinology.

[bib14] Drescher V.M., Gantt H.W., Whitehead W.E. (1980). Heart rate response to touch1. Psychosom. Med..

[bib15] Drescher V.M., Whitehead W.E., Morrill‐Corbin E.D., Cataldo M.F. (1985). Physiological and subjective reactions to being touched. Psychophysiology.

[bib16] Eckstein M., Mamaev I., Ditzen B., Sailer U. (2020). Calming effects of touch in human, animal, and robotic interaction—scientific state-of-the-art and technical advances. Front. Psychiatr..

[bib17] Erlichman J., Kerbey A.L., James W.P.T. (2002). Physical activity and its impact on health outcomes. Paper 1: the impact of physical activity on cardiovascular disease and all‐cause mortality: an historical perspective. Obes. Rev..

[bib18] Ferrell-Torry A.T., Glick O.J. (1993). The use of therapeutic massage as a nursing intervention to modify anxiety and the perception of cancer pain. Cancer Nurs..

[bib21] Field T. (2010). Touch for socioemotional and physical well-being: a review. Dev. Rev..

[bib20] Field T. (2016). Massage therapy research review. Compl. Ther. Clin. Pract..

[bib22] Field T., Diego M., Hernandez-Reif M., Schanberg S., Kuhn C. (2004). Massage therapy effects on depressed pregnant women. J. Psychosom. Obstet. Gynecol..

[bib19] Field T.M. (1998). Massage therapy effects. Am. Psychol..

[bib23] Floyd K. (2014). Relational and health correlates of affection deprivation. West. J. Commun..

[bib24] Floyd K., Boren J.P., Hannawa A.F., Hesse C., McEwan B., Veksler A.E. (2009). Kissing in marital and cohabiting relationships: effects on blood lipids, stress, and relationship satisfaction. West. J. Commun..

[bib25] Floyd K., Hesse C., Haynes M.T. (2007). Human affection exchange: XV. Metabolic and cardiovascular correlates of trait expressed affection. Commun. Q..

[bib26] Floyd K., Ray C.D., Raalte L.v., Stein J.B., Generous M.A. (2018). Interpersonal touch buffers pain sensitivity in romantic relationships but heightens sensitivity between strangers and friends. Research in Psychology and Behavioral Sciences.

[bib27] Goldman N., Turra C.M., Glei D.A., Seplaki C.L., Lin Y.-H., Weinstein M. (2006). Predicting mortality from clinical and nonclinical biomarkers. J. Gerontol., Ser. A Biol. Sci. Med. Sci..

[bib28] Grewen K.M., Anderson B.J., Girdler S.S., Light K.C. (2003). Warm partner contact is related to lower cardiovascular reactivity. Behav. Med..

[bib29] Guerrero L.K. (1997). Nonverbal involvement across interactions with same-sex friends, opposite-sex friends and romantic partners: consistency or change?. J. Soc. Pers. Relat..

[bib30] Guest S., Mehrabyan A., Ackerley R., McGlone F., Phillips N., Essick G. (2014). Tactile experience does not ameliorate age-related reductions in sensory function. Exp. Aging Res..

[bib31] Harlow H.F. (1958). The nature of love. Am. Psychol..

[bib32] Hawkley L.C., Kocherginsky M., Wong J., Kim J., Cagney K.A. (2014). Missing data in wave 2 of NSHAP: prevalence, predictors, and recommended treatment. J. Gerontol. B Psychol. Sci. Soc. Sci..

[bib33] Heatley Tejada A., Dunbar R., Montero M. (2020). Physical contact and loneliness: being touched reduces perceptions of loneliness. Adaptive human behavior and physiology.

[bib34] Heidinger T., Richter L. (2020). The effect of COVID-19 on loneliness in the elderly. An empirical comparison of pre-and peri-pandemic loneliness in community-dwelling elderly. Front. Psychol..

[bib35] Herman J.P., Nawreen N., Smail M.A., Cotella E.M. (2020). Brain mechanisms of HPA axis regulation: neurocircuitry and feedback in context richard kvetnansky lecture. Stress.

[bib36] Holt-Lunstad J., Birmingham W.A., Light K.C. (2008). Influence of a "warm touch" support enhancement intervention among married couples on ambulatory blood pressure, oxytocin, alpha amylase, and cortisol. Psychosom. Med..

[bib37] House J.S., Landis K.R., Umberson D. (1988). Social relationships and health. Science.

[bib38] Jolink T.A., Way B.M., Younge A., Oveis C., Algoe S.B. (2023). Everyday co-presence with a romantic partner is associated with lower C-reactive protein. Brain Behav. Immun..

[bib39] Kane H.S., McCall C., Collins N.L., Blascovich J. (2012). Mere presence is not enough: responsive support in a virtual world. J. Exp. Soc. Psychol..

[bib40] Karlamangla A.S., Singer B.H., McEwen B.S., Rowe J.W., Seeman T.E. (2002). Allostatic load as a predictor of functional decline: Macarthur studies of successful aging. J. Clin. Epidemiol..

[bib41] Kreuder A.K., Wassermann L., Wollseifer M., Ditzen B., Eckstein M., Stoffel-Wagner B., Hennig J., Hurlemann R., Scheele D. (2019). Oxytocin enhances the pain-relieving effects of social support in romantic couples. Hum. Brain Mapp..

[bib42] Lee J.E., Cichy K.E. (2020). Complex role of touch in social relationships for older adults' cardiovascular disease risk. Res. Aging.

[bib43] Light K.C., Grewen K.M., Amico J.A. (2005). More frequent partner hugs and higher oxytocin levels are linked to lower blood pressure and heart rate in premenopausal women. Biol. Psychol..

[bib44] Lindgren L., Rundgren S., Winsö O., Lehtipalo S., Wiklund U., Karlsson M., Stenlund H., Jacobsson C., Brulin C. (2010). Physiological responses to touch massage in healthy volunteers. Auton. Neurosci..

[bib45] Liu D., Piao Y., Ma R., Zhang Y., Guo W., Zuo L., Liu W., Song H., Zhang X. (2021). Actor and partner effects of touch: touch-Induced stress alleviation is influenced by perceived relationship quality of the couple. Front. Psychol..

[bib46] Lynch J.J., Flaherty L., Emrich C., Mills M.E., Katcher A. (1974). Effects of human contact on the heart activity of curarized patients in a shock-trauma unit. Am. Heart J..

[bib47] McDade T.W., Burhop J., Dohnal J. (2004). High-sensitivity enzyme immunoassay for C-reactive protein in dried blood spots. Clin. Chem..

[bib48] McEwen B.S. (1998). Stress, adaptation, and disease: allostasis and allostatic load. Ann. N. Y. Acad. Sci..

[bib49] McEwen B.S. (2013). Allostasis and allostatic load: implications for neuropsychopharmacology. Stress and the Brain.

[bib50] McEwen B.S., Akil H. (2020). Revisiting the stress concept: implications for affective disorders. J. Neurosci..

[bib51] Morrison I. (2016). Keep calm and cuddle on: social touch as a stress buffer. Adaptive Human Behavior and Physiology.

[bib52] Morriss J., Bell T., Johnstone T., Van Reekum C.M., Hill J. (2019). Social domain based modulation of neural responses to threat: the different roles of romantic partners versus friends. Soc. Neurosci..

[bib53] Murphy M.L., Janicki-Deverts D., Cohen S. (2018). Receiving a hug is associated with the attenuation of negative mood that occurs on days with interpersonal conflict. PLoS One.

[bib54] Navyte G., Gillmeister H., Kumari M. (2024). Interpersonal touch and the importance of romantic partners for older adults' neuroendocrine health. Psychoneuroendocrinology.

[bib55] ONS (2021). *Families and households in the UK: 2020* office for national Statistics. https://www.ons.gov.uk/peoplepopulationandcommunity/birthsdeathsandmarriages/families/bulletins/familiesandhouseholds/2020.

[bib56] Packheiser J., Hartmann H., Fredriksen K., Gazzola V., Keysers C., Michon F. (2023). The physical and mental health benefits of touch interventions: a comparative systematic review and multivariate meta-analysis. medRxiv.

[bib57] Packheiser J., Hartmann H., Fredriksen K., Gazzola V., Keysers C., Michon F. (2024). A systematic review and multivariate meta-analysis of the physical and mental health benefits of touch interventions. Nat. Hum. Behav..

[bib58] Packheiser J., Malek I.M., Reichart J.S., Katona L., Luhmann M., Ocklenburg S. (2022). The association of embracing with daily mood and general life satisfaction: an ecological momentary assessment study. J. Nonverbal Behav..

[bib59] Packheiser J., Sommer L., Wüllner M., Malek I.M., Reichart J.S., Katona L. (2024). A comparison of hugging frequency and its association with momentary mood before and during COVID-19 using ecological momentary assessment. Health Commun..

[bib60] Palgi Y., Shrira A., Ring L., Bodner E., Avidor S., Bergman Y., Cohen-Fridel S., Keisari S., Hoffman Y. (2020). The loneliness pandemic: loneliness and other concomitants of depression, anxiety and their comorbidity during the COVID-19 outbreak. J. Affect. Disord..

[bib61] Pedell S., Vetere F., Kulik L., Ozanne E., Gruner A. (2010). Proceedings of the 22nd Conference of the Computer-Human Interaction Special Interest Group of Australia on Computer-Human Interaction.

[bib62] Pino L., González‐Vélez A.E., Prieto‐Flores M.E., Ayala A., Fernandez‐Mayoralas G., Rojo‐Perez F., Martinez‐Martin P., Forjaz M.J. (2014). Self‐perceived health and quality of life by activity status in community‐dwelling older adults. Geriatr. Gerontol. Int..

[bib63] Roberts M.H., Klatzkin R.R., Mechlin B. (2015). Social support attenuates physiological stress responses and experimental pain sensitivity to cold pressor pain. Ann. Behav. Med..

[bib64] Robinson K.J., Hoplock L.B., Cameron J.J. (2015). When in doubt, reach out: touch is a covert but effective mode of soliciting and providing social support. Soc. Psychol. Personal. Sci..

[bib65] Scharf T., de Jong Gierveld J. (2008). Loneliness in urban neighbourhoods: an anglo-dutch comparison. Eur. J. Ageing.

[bib66] Schneider E., Hopf D., Aguilar-Raab C., Scheele D., Neubauer A.B., Sailer U. (2023). Affectionate touch and diurnal oxytocin levels: an ecological momentary assessment study. eLife.

[bib67] Schneider E., Hopf D., Eckstein M., Aguilar-Raab C., Scheele D., Hurlemann R., Ditzen B. (2021). Salivary oxytocin and touch in everyday life: results from an EMA study during Covid-19 lockdown. Psychoneuroendocrinology.

[bib70] Sedgwick P. (2012). What is recall bias?. Br. Med. J..

[bib68] Seeman T.E. (1996). Social ties and health: the benefits of social integration. Ann. Epidemiol..

[bib69] Seeman T.E., McEwen B.S., Rowe J.W., Singer B.H. (2001). Allostatic load as a marker of cumulative biological risk: Macarthur studies of successful aging. Proc. Natl. Acad. Sci..

[bib71] Sehlstedt I., Ignell H., Backlund Wasling H., Ackerley R., Olausson H., Croy I. (2016). Gentle touch perception across the lifespan. Psychol. Aging.

[bib72] Shaltout H.A., Tooze J.A., Rosenberger E., Kemper K.J. (2012). Time, touch, and compassion: effects on autonomic nervous system and well-being. Explore.

[bib73] Shaver P., Hazan C. (1987). Being lonely, falling in love. J. Soc. Behav. Pers..

[bib75] Sorokowska A., Kowal M., Saluja S., Aavik T., Alm C., Anjum A. (2023). Love and affectionate touch toward romantic partners all over the world. Sci. Rep..

[bib74] Sorokowska A., Saluja S., Sorokowski P., Frąckowiak T., Karwowski M., Aavik T. (2021). Affective interpersonal touch in close relationships: a cross-cultural perspective. Pers. Soc. Psychol. Bull..

[bib76] Stein N., Sanfilipo M. (1985). Depression and the wish to be held. J. Clin. Psychol..

[bib77] Sterling P., Eyer J. (1988). Handbook of Life Stress, Cognition and Health’.

[bib78] Tang Y., Benusiglio D., Lefevre A., Hilfiger L., Althammer F., Bludau A., Hagiwara D., Baudon A., Darbon P., Schimmer J., Kirchner M.K., Roy R.K., Wang S., Eliava M., Wagner S., Oberhuber M., Conzelmann K.K., Schwarz M., Stern J.E., Leng G., Neumann I.D., Charlet A., Grinevich V. (2020). Social touch promotes interfemale communication via activation of parvocellular oxytocin neurons. Nat. Neurosci..

[bib79] Thomas P.A., Kim S. (2021). Lost touch? Implications of physical touch for physical health. J. Gerontol.: Ser. Bibliogr..

[bib80] Triscoli C., Croy I., Olausson H., Sailer U. (2017). Touch between romantic partners: being stroked is more pleasant than stroking and decelerates heart rate. Physiol. Behav..

[bib81] Uchino B.N. (2004).

[bib82] Uchino B.N. (2006). Social support and health: a review of physiological processes potentially underlying links to disease outcomes. J. Behav. Med..

[bib83] Uvnäs-Moberg K. (1998). Oxytocin May mediate the benefits of positive social interaction and emotions. Psychoneuroendocrinology.

[bib84] Van Puyvelde M., Staring L., Schaffers J., Rivas‐Smits C., Groenendijk L., Smeyers L. (2021). Why do we hunger for touch? The impact of daily gentle touch stimulation on maternal‐infant physiological and behavioral regulation and resilience. Infant Ment. Health J..

[bib85] Van Raalte L.J., Floyd K., Mongeau P.A. (2021). The effects of cuddling on relational quality for married couples: a longitudinal investigation. West. J. Commun..

[bib86] Von Mohr M., Kirsch L.P., Fotopoulou A. (2017). The soothing function of touch: affective touch reduces feelings of social exclusion. Sci. Rep..

[bib87] Von Mohr M., Kirsch L.P., Fotopoulou A. (2021). Social touch deprivation during COVID-19: effects on psychological wellbeing and craving interpersonal touch. R. Soc. Open Sci..

[bib88] Wills T.A., Clark M.S. (1991). Prosocial Behavior.

[bib89] Yang Y.C., Boen C., Gerken K., Li T., Schorpp K., Harris K.M. (2016). Social relationships and physiological determinants of longevity across the human life span. Proc. Natl. Acad. Sci..

