# Supplementary material for: Long-term metabolic and cardiovascular health benefits from romantic partner touch in older adults
Source: Brain Behav Immun Health. 2026 Sep 9;57:101352. doi: 10.1016/j.bbih.2026.101352 (PMC13583967; doi:10.1016/j.bbih.2026.101352)
Supplement: Supplementary file 1 [file mmc1.docx]

**Table A.1.** *Descriptive statistics of respondents in the analytic sample versus those not in the analytic sample by sociodemographic characteristics and covariates*

| Sociodemographic characteristics and covariates | Present in analytic sample (n=787) | Not present in analytic sample (n=1,474) |
| --- | --- | --- |
|  | *Frequency (%)* | *Frequency (%)* |
| Sex |  |  |
| Men | 51.70 | 45.90 |
| Women | 48.30 | 54.10 |
| Age |  |  |
| Wave 1 |  |  |
| 57-64 | 37.10 | 38.30 |
| 65-74 | 40.20 | 36.10 |
| 75-85 | 22.70 | 25.60 |
| Wave 2 |  |  |
| 62-69 | 36.20 | 37.90 |
| 70-79 | 40.80 | 36.20 |
| 80-91 | 23.00 | 26.00 |
| Educational Attainment* |  |  |
| < High school degree | 18.30 | 21.20 |
| High school degree or equivalent | 24.10 | 25.60 |
| Some college or associate degree | 32.10 | 29.60 |
| Bachelor’s degree or higher | 25.40 | 23.60 |
| Total Household Assets*^a^ |  |  |
| 0-49,999 | 16.00 | 17.80 |
| 50,000-99,999 | 9.15 | 6.72 |
| 100,000-499,999 | 33.50 | 29.20 |
| 500k or higher | 26.00 | 22.30 |
| Ethnicity*^b^ |  |  |
| White | 73.10 | 69.10 |
| Non-White | 24.40 | 28.20 |
| Romantic Partner* |  |  |
| Yes | 70.30 | 71.50 |
| No | 29.70 | 28.50 |
| Number of co-residents* |  |  |
| Lives alone | 24.90 | 25.30 |
| Lives with one other person | 54.10 | 53.90 |
| Lives with two or more persons | 21.00 | 20.80 |
| Frequency of Sexual Touch *^c^ |  |  |
| No sex | 43.60 | 45.30 |
| Infrequent sexual touch | 11.70 | 11.90 |
| Frequent sexual touch | 42.90 | 39.60 |
| Alcohol Intake*^d^ |  |  |
| Regular drinkers | 43.60 | 40.80 |
| Non-regular drinkers | 23.50 | 22.00 |
| Never drank alcohol | 16.30 | 20.80 |
| Frequency of Touch with Partner |  |  |
| Wave 1^e^ |  |  |
| >Weekly | 51.80 | 51.50 |
| Weekly | 5.97 | 7.80 |
| Monthly | 7.50 | 5.83 |
| Never | 1.02 | 1.15 |
| Wave 2^f^ |  |  |
| >Weekly | 39.40 | 34.90 |
| Weekly | 5.08 | 5.90 |
| Monthly | 5.97 | 6.17 |
| Never | 2.80 | 2.78 |
| Frequency of Touch with Others |  |  |
| Wave 1^g^ |  |  |
| >Weekly | 20.10 | 21.30 |
| Weekly | 21.10 | 18.50 |
| Monthly | 28.30 | 28.30 |
| Never | 24.30 | 24.90 |
| Wave 2^h^ |  |  |
| >Weekly | 42.90 | 40.60 |
| Weekly | 20.10 | 19.00 |
| Monthly | 19.10 | 18.20 |
| Never | 6.23 | 5.43 |

*Note.* *Responses were used from Wave 1 and if not available, from Wave 2

^a^ 120 (15.20%) respondents in the analytic sample and 354 (24.00%) in the non-analytic sample had incomplete responses for total household assets

^b^ 20 (2.54%) respondents in the analytic sample and 41 (2.78%) in the non-analytic sample had incomplete responses for ethnicity

^c^ 14 (1.78%) respondents in the analytic sample and 48 (3.26%) in the non-analytic sample had incomplete responses for frequency of sexual touch

^d^ 131 (16.60%) respondents in the analytic sample and 240 (16.30%) in the non-analytic sample had incomplete responses for alcohol intake

^e^ 265 (33.70%) respondents in the analytic sample and 497 (33.70%) in the non-analytic sample had incomplete responses for frequency of touch with a romantic partner at Wave 1

^f^ 368 (46.8%) respondents in the analytic sample and 741 (50.30%) in the non-analytic sample had incomplete responses for frequency of touch with a romantic partner at Wave 2

^g^ 49 (6.23%) respondents in the analytic sample and 103 (6.99%) in the non-analytic sample had incomplete responses for frequency of touch with other close adults at Wave 1

^h^ 92 (11.70%) respondents in the analytic sample and 247 (16.80%) in the non-analytic sample had incomplete responses for frequency of touch with other close adults at Wave 2

**Table A.2.** *Prevalence of antihypertensive and antidiabetic medication use by demographic and lifestyle covariates across NSHAP Waves 1 and 2.*

| Covariates | Antihypertensive (%) | | Antidiabetic (%) | |
| --- | --- | --- | --- | --- |
|  | Wave 1 | Wave 2 | Wave 1 | Wave 2 |
| Sex |  |  |  |  |
| Women | 10.00 | 13.90 | 17.40 | 18.40 |
| Men | 9.34 | 12.00 | 17.90 | 21.10 |
| Ethnicity |  |  |  |  |
| White | 8.87 | 11.50 | 14.40 | 16.50 |
| Non-White | 11.50 | 17.70 | 27.10 | 29.70 |
| Age (y) |  |  |  |  |
| 57-64 | 8.22 |  | 14.70 |  |
| 65-74 | 9.49 |  | 19.30 |  |
| 75-85 | 12.30 |  | 19.60 |  |
| 62-69 |  | 12.60 |  | 18.60 |
| 70-79 |  | 14.00 |  | 19.90 |
| 80-91 |  | 11.60 |  | 21.50 |
| Educational Attainment |  |  |  |  |
| <High School | 11.80 | 10.40 | 25.00 | 24.30 |
| High School Degree or Equivalent | 12.10 | 14.20 | 16.30 | 20.50 |
| Some College or Associate Degree | 7.91 | 14.20 | 19.80 | 21.30 |
| Bachelor’s Degree or Higher | 8.00 | 12.00 | 11.00 | 14.00 |
| Total Household Assets ($) |  |  |  |  |
| 0-49,999 | 7.22 | 11.40 | 23.70 | 21.90 |
| 50,000-99,999 | 11.70 | 13.60 | 23.30 | 37.30 |
| 100,000-499,999 | 5.91 | 12.40 | 18.60 | 16.70 |
| 500k or higher | 9.84 | 15.90 | 11.50 | 13.40 |
| Number of co-residents |  |  |  |  |
| Lives Alone | 10.20 | 13.40 | 18.40 | 18.0 |
| Lives with One Other Person | 8.22 | 13.40 | 16.90 | 18.30 |
| Lives with Two or More Persons | 12.80 | 11.20 | 18.90 | 26.10 |
| Romantic Partner |  |  |  |  |
| Yes | 9.95 | 13.70 | 16.30 | 18.1 |
| No | 8.97 | 11.70 | 20.90 | 23.0 |
| Frequency of Sexual Touch |  |  |  |  |
| No Sex | 8.73 | 13.50 | 21.70 | 20.60 |
| Infrequent Sexual Touch | 13.30 | 15.50 | 15.60 | 20.70 |
| Frequent Sexual Touch | 8.96 | 16.20 | 13.70 | 14.60 |
| Alcohol Intake |  |  |  |  |
| Never Drank | 13.70 | 14.30 | 25.30 | 31.20 |
| Non-Regular Drinker | 9.70 | 9.22 | 20.00 | 15.60 |
| Regular Drinker | 8.90 | 13.40 | 11.00 | 13.70 |

*Note.* Values represent the percentage of respondents taking antihypertensive or antidiabetic medication in Waves 1 and 2 of NSHAP.

**Table A.3.** *The relationship between touch frequency (from romantic partners and other adults), sociodemographic variables and covariates in the final sample (n = 787) from NSHAP Wave 1 (2005 to 2006) and 2 (2010 to 2011)*

| Demographic Characteristic | Wave | Partner Touch Frequency (%) | | | | Touch with Others Frequency (%) | | | |
| --- | --- | --- | --- | --- | --- | --- | --- | --- | --- |
|  |  | Never | Monthly | Weekly | >Weekly | Never | Monthly | Weekly | >Weekly |
| Sex |  |  |  |  |  |  |  |  |  |
| Women^a^ | 1 | 50.00 | 38.98 | 42.55 | 35.54 | 37.17 | 42.60 | 60.24 | 60.76 |
|  | 2 | 68.18 | 38.30 | 30.00 | 33.55 | 30.61 | 38.67 | 53.80 | 54.73 |
| Ethnicity |  |  |  |  |  |  |  |  |  |
| White^b^ | 1 | 25.00 | 62.07 | 75.56 | 85.18 | 70.59 | 82.03 | 77.78 | 74.03 |
|  | 2 | 68.18 | 73.33 | 71.79 | 85.43 | 57.14 | 85.03 | 83.66 | 75.23 |
| Age (y) |  |  |  |  |  |  |  |  |  |
| 57-64 | 1 | 37.50 | 38.98 | 42.55 | 43.38 | 34.55 | 40.81 | 35.54 | 36.71 |
| 65-74 | 1 | 37.50 | 37.29 | 44.68 | 38.48 | 41.88 | 35.87 | 37.95 | 45.57 |
| 75-85 | 1 | 25.00 | 23.73 | 12.77 | 18.14 | 23.56 | 23.32 | 26.51 | 17.72 |
| 62-69 | 2 | 45.45 | 48.94 | 45.00 | 42.58 | 28.57 | 40.00 | 32.91 | 37.57 |
| 70-79 | 2 | 31.82 | 44.68 | 32.50 | 41.29 | 42.86 | 36.67 | 37.97 | 44.38 |
| 80-91 | 2 | 22.73 | 6.38 | 22.50 | 16.13 | 28.57 | 23.33 | 29.11 | 18.05 |
|  |  | Never | Monthly | Weekly | >Weekly | Never | Monthly | Weekly | >Weekly |
| Educational Attainment |  |  |  |  |  |  |  |  |  |
| <High School | 1 | 37.50 | 23.73 | 10.64 | 12.99 | 18.85 | 14.80 | 16.27 | 18.99 |
|  | 2 | 13.64 | 14.89 | 15.00 | 13.23 | 32.65 | 13.33 | 11.39 | 18.64 |
| High School Degree or Equivalent | 1 | 12.50 | 32.20 | 29.79 | 24.75 | 30.89 | 26.46 | 18.07 | 21.52 |
|  | 2 | 36.36 | 23.40 | 27.50 | 23.55 | 28.57 | 28.67 | 25.32 | 21.89 |
| Some College or Associate Degree | 1 | 37.50 | 32.20 | 25.53 | 32.35 | 32.46 | 29.15 | 36.14 | 33.54 |
|  | 2 | 36.36 | 36.17 | 35.00 | 32.58 | 26.53 | 27.33 | 39.87 | 32.25 |
| Bachelor’s Degree or Higher | 1 | 12.50 | 11.86 | 34.04 | 29.90 | 17.80 | 29.60 | 29.52 | 25.95 |
|  | 2 | 13.64 | 25.53 | 22.50 | 30.65 | 12.24 | 30.67 | 23.42 | 27.22 |
| Total Household Assets ($)^c^ |  |  |  |  |  |  |  |  |  |
| 0-49,999 | 2 | 50.00 | 10.87 | 11.43 | 9.22 | 19.26 | 15.03 | 8.33 | 22.43 |
| 50,000-99,999 | 2 | 16.67 | 8.70 | 5.71 | 7.45 | 10.37 | 8.67 | 12.04 | 13.08 |
| 100,000-499,999 | 2 | 16.67 | 54.35 | 42.86 | 37.94 | 36.30 | 45.09 | 42.59 | 31.78 |
| 500k or higher | 2 | 16.67 | 26.09 | 40.00 | 45.39 | 34.07 | 31.21 | 37.04 | 32.71 |
| Number of co-residents |  |  |  |  |  |  |  |  |  |
| Lives Alone | 1 | 0.00 | 6.78 | 4.26 | 6.63 | 21.47 | 27.48 | 25.90 | 24.68 |
|  | 2 | 4.55 | 0.00 | 0.00 | 0.32 | 20.41 | 20.00 | 29.75 | 20.71 |
| Lives with One Other Person | 1 | 87.50 | 67.80 | 74.47 | 74.45 | 60.21 | 55.41 | 54.22 | 51.27 |
|  | 2 | 72.73 | 76.60 | 75.00 | 83.23 | 44.90 | 66.00 | 54.43 | 48.52 |
| Lives with Two or More Persons | 1 | 12.50 | 25.42 | 21.28 | 18.92 | 18.32 | 17.12 | 19.88 | 24.05 |
|  | 2 | 22.73 | 23.40 | 25.00 | 16.45 | 34.69 | 14.00 | 15.82 | 20.71 |
|  |  | Never | Monthly | Weekly | >Weekly | Never | Monthly | Weekly | >Weekly |
| Romantic Partner^d^ |  |  |  |  |  |  |  |  |  |
| Yes | 1 |  |  |  |  | 74.87 | 69.06 | 69.28 | 68.35 |
|  | 2 |  |  |  |  | 55.10 | 73.33 | 65.19 | 62.13 |
| Frequency of Sexual Touch |  |  |  |  |  |  |  |  |  |
| No Sex | 1 | 62.50 | 41.82 | 37.21 | 20.87 | 46.45 | 41.78 | 41.72 | 47.02 |
|  | 2 | 78.95 | 56.82 | 30.56 | 28.31 | 36.36 | 42.86 | 34.83 | 33.33 |
| Infrequent Sexual Touch | 1 | 25.00 | 25.45 | 23.26 | 11.96 | 11.48 | 13.62 | 8.59 | 11.92 |
|  | 2 | 10.53 | 22.73 | 33.33 | 12.50 | 9.09 | 18.37 | 19.10 | 11.70 |
| Frequent Sexual Touch | 1 | 12.50 | 32.73 | 39.53 | 67.18 | 42.08 | 44.60 | 49.69 | 41.06 |
|  | 2 | 10.53 | 20.45 | 36.11 | 59.19 | 54.55 | 38.78 | 46.07 | 54.97 |
| Alcohol Intake |  |  |  |  |  |  |  |  |  |
| Never Drank | 1 | 14.29 | 18.60 | 13.51 | 9.84 | 16.80 | 11.05 | 15.27 | 23.01 |
|  | 2 | 21.05 | 23.08 | 16.67 | 15.58 | 24.24 | 10.38 | 16.13 | 22.76 |
| Non-Regular Drinker | 1 | 57.14 | 23.26 | 29.73 | 26.56 | 39.20 | 26.52 | 25.95 | 23.89 |
|  | 2 | 15.79 | 30.77 | 6.67 | 22.51 | 27.27 | 26.42 | 28.23 | 24.80 |
| Regular Drinker | 1 | 28.57 | 58.14 | 56.76 | 63.61 | 44.00 | 62.43 | 58.78 | 53.10 |
|  | 2 | 63.16 | 46.15 | 76.67 | 61.90 | 48.48 | 63.21 | 55.65 | 52.44 |

*Note.* The percentages represent the distribution of touch frequency within each demographic group. For example, among those who reported “Never” engaging in partner touch, 14.29% were non-drinkers, 57.14% were non-regular drinkers, and 28.57% were regular drinkers. Comparisons are made between the listed categories within each demographic group. Groups without a direct comparison in the table (such as sex, ethnicity, romantic partner status) are clarified in the note below. N’s for each touch group are not reported as FIML was used.

^a^Women compared to men

^b^White respondents compared to black and Hispanic respondents

^c^Only measured at Wave 2

^d^Respondents with a romantic partner compared to no romantic partner

**Table A.4.** *Spearman correlations between all Wave 1 measures*

|  |  | Other close adult touch frequency | Waist circumference | Log CRP | Log HbA1c | BMI | sBP | dBP | Pulse | Log DHEA | Age | Highest qualification | Household assets | Household composition | Sexual touch | Alcohol intake |
| --- | --- | --- | --- | --- | --- | --- | --- | --- | --- | --- | --- | --- | --- | --- | --- | --- |
| Romantic partner touch frequency | | 0.23******* | -0.04 | -0.11****** | -0.01 | -0.05 | 0.03 | 0.02 | -0.13****** | -0.04 | -0.03 | 0.02 | 0.14****** | -0.04 | -0.27******* | 0.09 |
| Other close adult touch frequency | | | -0.11****** | 0.00 | 0.00 | 0.02 | -0.07 | 0.01 | 0.01 | 0.00 | -0.02 | -0.02 | 0.02 | 0.01 | -0.01 | 0.02 |
| Waist circumference | | | | 0.24******* | 0.28******* | 0.78******* | 0.07 | 0.11****** | 0.06 | 0.02 | -0.04 | -0.05 | -0.06 | 0.07***** | -0.04 | -0.02 |
| Log CRP | | | | | 0.13******* | 0.30******* | 0.07***** | 0.10****** | 0.18******* | 0.03 | -0.02 | -0.04 | -0.09***** | -0.03 | 0.14******* | -0.14******* |
| Log HbA1c | | | | | | 0.24******* | 0.04 | -0.06 | 0.08***** | -0.02 | 0.06 | -0.05 | -0.18******* | 0.02 | 0.13******* | -0.18******* |
| BMI | | | | | | | 0.02 | 0.17******* | 0.12******* | 0.06 | -0.11****** | -0.03 | -0.12****** | 0.04 | 0.07***** | -0.11****** |
| sBP | | | | | | | | 0.57******* | -0.08***** | 0.00 | 0.06 | -0.02 | -0.10***** | -0.06 | 0.02 | 0.03 |
| dBP | | | | | | | | | 0.16******* | 0.13******* | -0.24******* | 0.05 | -0.07 | -0.04 | -0.03 | 0.04 |
| Pulse | | | | | | | | | | 0.09***** | -0.06 | 0.02 | -0.15******* | -0.05 | 0.06 | -0.04 |
| Log DHEA | | | | | | | | | | | -0.26******* | 0.00 | -0.03 | 0.09****** | -0.12******* | 0.09***** |
| Age | | | | | | | | | | | | -0.05 | -0.05 | -0.17******* | 0.32******* | -0.11****** |
| Highest Qualification | | | | | | | | | | | | | 0.10***** | -0.02 | -0.05 | 0.11****** |
| Household assets | | | | | | | | | | | | | | 0.08***** | -0.17******* | 0.28******* |
| Household composition | | | | | | | | | | | | | | | -0.22******* | 0.01 |
| Sexual touch | | | | | | | | | | | | | | | | 0.22*** |

*Note.* Values are Spearman correlation coefficients. * p < .05, ** p < .01, *** p < .001.

**Table A.5.** *Full parameter estimates (B, 95% CI, and p-values) for all predictors, including covariates, in the final cross-lagged models predicting Wave 2 metabolic, cardiovascular, and neuroendocrine health and touch frequency (NSHAP Waves 1-2, n = 787).*

| *Outcome* | *Predictor* | *Covariates* | *B* | 95% CI |
| --- | --- | --- | --- | --- |
| *Wave 2 metabolic health* |  |  |  |  |
|  | *Wave 1 metabolic health* |  | *1.00*** | *0.95 – 1.06* |
|  | Wave 1 romantic partner touch frequency |  | *-0.08*** | *(-0.13 - -0.02)* |
|  |  | *Age* | *-0.09*** | *-0.14 - -0.04* |
|  |  | *Female* | *0.15*** | *0.05 – 0.24* |
|  |  | *Ethnicity* | *0.09* | *-0.01 – 0.18* |
|  |  | *Highest qualification* | *-0.01* | *-0.04 – 0.02* |
|  |  | *Total household assets* | *0.03* | *-0.02 – 0.07* |
|  |  | *Household composition* | *0.04* | *-0.01 – 0.10* |
|  |  | *Partnered* | *-0.11** | *-0.21 - -0.01* |
|  |  | *Sexual touch* | *-0.03* | *-0.07 – 0.02* |
|  |  | *Alcohol consumption* | *-0.05* | *-0.10 – 0.01* |
|  | Wave 1 other adult touch frequency |  | *-0.00* | *-0.03 – 0.03* |
|  |  | *Age* | *-0.09*** | *-0.14 - -0.05* |
|  |  | *Female* | *0.15*** | *0.05 – 0.24* |
|  |  | *Ethnicity* | *0.06* | *-0.03 – 0.15* |
|  |  | *Highest qualification* | *-0.01* | *-0.04 – 0.03* |
|  |  | *Total household assets* | *0.02* | *-0.02 – 0.06* |
|  |  | *Household composition* | *0.04* | *-0.01 – 0.10* |
|  |  | *Partnered* | *-0.09* | *-0.19 – 0.01* |
|  |  | *Sexual touch* | *-0.01* | *-0.06 – 0.03* |
|  |  | *Alcohol consumption* | *-0.05* | *-0.11 – 0.01* |
| *Wave 2 cardiovascular health* |  |  |  |  |
|  | *Wave 1 cardiovascular health* |  | *0.60*** | *0.51 – 0.69* |
|  | *Wave 1 romantic partner touch frequency* |  | *-0.14*** | *-0.23 - -0.04* |
|  |  | *Age* | *-0.05* | *-0.13 – 0.03* |
|  |  | *Female* | *-0.04* | *-0.17 – 0.08* |
|  |  | *Ethnicity* | *0.11* | *-0.05 – 0.27* |
|  |  | *Highest qualification* | *-0.04* | *-0.09 – 0.02* |
|  |  | *Total household assets* | *-0.05* | *-0.11 – 0.02* |
|  |  | *Household composition* | *0.08* | *-0.01 – 0.17* |
|  |  | *Partnered* | *-0.17* | *-0.34 - -0.00* |
|  |  | *Sexual touch* | *-0.06* | *-0.14 – 0.02* |
|  |  | *Alcohol consumption* | *-0.02* | *-0.11 – 0.08* |
|  | *Wave 1 other adult touch frequency* |  | *0.01* | *-0.05 – 0.06* |
|  |  | *Age* | *-0.06* | *-0.14 – 0.03* |
|  |  | *Female* | *-0.04* | *-0.17 – 0.09* |
|  |  | *Ethnicity* | *0.06* | *-0.10 – 0.21* |
|  |  | *Highest qualification* | *-0.03* | *-0.08 – 0.02* |
|  |  | *Total household assets* | *-0.05* | *-0.12 – 0.01* |
|  |  | *Household composition* | *0.07* | *-0.02 – 0.17* |
|  |  | *Partnered* | *-0.13* | *-0.30 – 0.03* |
|  |  | *Sexual touch* | *-0.03* | *-0.11 – 0.04* |
|  |  | *Alcohol consumption* | *-0.02* | *-0.12 – 0.07* |
| Wave 2 neuroendocrine health |  |  |  |  |
|  | *Wave 1 neuroendocrine health* |  | *1.57*** | *0.55 – 2.58* |
|  | *Wave 1 romantic partner touch frequency* |  | *0.04* | *-0.04 – 0.12* |
|  |  | *Age* | *0.03* | *-0.03 – 0.10* |
|  |  | *Female* | *0.05* | *-0.06 – 0.15* |
|  |  | *Ethnicity* | *-0.02* | *-0.16 – 0.11* |
|  |  | *Highest qualification* | *-0.01* | *-0.06 – 0.03* |
|  |  | *Total household assets* | *-0.04* | *-0.10 – 0.01* |
|  |  | *Household composition* | *0.07* | *-0.01 – 0.15* |
|  |  | *Partnered* | *-0.08* | *-0.22 – 0.06* |
|  |  | *Sexual touch* | *0.03* | *-0.04 – 0.10* |
|  |  | *Alcohol consumption* | *-0.03* | *-0.11 – 0.06* |
|  | *Wave 1 other adult touch frequency* |  | *0.03* | *-0.02 – 0.07* |
|  |  | *Age* | *0.04* | *-0.03 – 0.10* |
|  |  | *Female* | *0.06* | *-0.05 – 0.17* |
|  |  | *Ethnicity* | *-0.01* | *-0.14 – 0.12* |
|  |  | *Highest qualification* | *-0.01* | *-0.06 – 0.03* |
|  |  | *Total household assets* | *-0.04* | *-0.10 – 0.02* |
|  |  | *Household composition* | *0.07* | *-0.01 – 0.15* |
|  |  | *Partnered* | *-0.09* | *-0.23 – 0.05* |
|  |  | *Sexual touch* | *0.02* | *-0.04 – 0.09* |
|  |  | *Alcohol consumption* | *-0.03* | *-0.11 – 0.05* |
| Wave 2 romantic partner touch frequency |  |  |  |  |
|  | Wave 1 romantic partner touch frequency |  | *0.63*** | *0.52 – 0.74* |
|  | Wave 1 metabolic health |  | *-0.00* | *-0.03 – 0.09* |
|  |  | *Age* | *0.08* | *-0.03 – 0.19* |
|  |  | *Female* | *0.13* | *-0.04 – 0.29* |
|  |  | *Ethnicity* | *0.20* | *-0.02 – 0.42* |
|  |  | *Highest qualification* | *-0.05* | *-0.12 – 0.02* |
|  |  | *Total household assets* | *-0.01* | *-0.10 – 0.07* |
|  |  | *Household composition* | *0.11* | *-0.06 – 0.27* |
|  |  | *Partnered* | *0.01* | *-0.61 – 0.63* |
|  |  | *Sexual touch* | *-0.05* | *-0.15 – 0.05* |
|  |  | *Alcohol consumption* | *0.06* | *-0.07 – 0.19* |
|  | Wave 1 cardiovascular health |  | *0.01* | *-0.10 – 0.12* |
|  |  | *Age* | *0.08* | *-0.02 – 0.19* |
|  |  | *Female* | *0.12* | *-0.04 – 0.28* |
|  |  | *Ethnicity* | *0.21* | *-0.02 – 0.43* |
|  |  | *Highest qualification* | *-0.05* | *-0.12 – 0.02* |
|  |  | *Total household assets* | *-0.01* | *-0.10 – 0.07* |
|  |  | *Household composition* | *0.11* | *-0.06 – 0.27* |
|  |  | *Partnered* | *-0.01* | *-0.62 – 0.61* |
|  |  | *Sexual touch* | *-0.06* | *-0.16 – 0.04* |
|  |  | *Alcohol consumption* | *0.06* | *-0.07 – 0.19* |
|  | *Wave 1 neuroendocrine health* |  | *0.12* | *-0.46 – 0.69* |
|  |  | *Age* | *0.09* | *-0.02 – 0.20* |
|  |  | *Female* | *0.12* | *-0.04 – 0.28* |
|  |  | *Ethnicity* | *0.20* | *-0.02 – 0.42* |
|  |  | *Highest qualification* | *-0.05* | *-0.12 – 0.02* |
|  |  | *Total household assets* | *-0.01* | *-0.10 – 0.07* |
|  |  | *Household composition* | *0.11* | *-0.06 – 0.27* |
|  |  | *Partnered* | *0.00* | *-0.62 – 0.63* |
|  |  | *Sexual touch* | *-0.06* | *-0.16 – 0.04* |
|  |  | *Alcohol consumption* | *0.06* | *-0.07 – 0.19* |
| Wave 2 other adult touch frequency |  |  |  |  |
|  | Wave 1 other adult touch frequency |  | *0.23*** | *0.17 – 0.30* |
|  | Wave 1 metabolic health |  | *0.07* | *-0.02 – 0.16* |
|  |  | *Age* | *-0.04* | *-0.14 – 0.06* |
|  |  | *Female* | *-0.23*** | *-0.40 - -0.06* |
|  |  | *Ethnicity* | *-0.02* | *-0.23 – 0.18* |
|  |  | *Highest qualification* | *0.01* | *-0.06 – 0.08* |
|  |  | *Total household assets* | *0.08* | *-0.01 – 0.16* |
|  |  | *Household composition* | *0.02* | *-0.10 – 0.14* |
|  |  | *Partnered* | *-0.14* | *-0.35 – 0.08* |
|  |  | *Sexual touch* | *-0.09* | *-0.35 – 0.08* |
|  |  | *Alcohol consumption* | *-0.05* | *-0.18 – 0.07* |
|  | *Wave 1 cardiovascular health* |  | *-0.02* | *-0.13 – 0.09* |
|  |  | *Age* | *-0.05* | *-0.15 – 0.05* |
|  |  | *Female* | *-0.19** | *-0.36 - -0.03* |
|  |  | *Ethnicity* | *-0.02* | *-0.22 – 0.19* |
|  |  | *Highest qualification* | *0.01* | *-0.06 – 0.08* |
|  |  | *Total household assets* | *0.07* | *-0.01 – 0.16* |
|  |  | *Household composition* | *0.02* | *-0.10 – 0.14* |
|  |  | *Partnered* | *-0.15* | *-0.36 – 0.07* |
|  |  | *Sexual touch* | *-0.08* | *-0.18 – 0.02* |
|  |  | *Alcohol consumption* | *-0.07* | *-0.19 – 0.06* |
|  | *Wave 1 neuroendocrine health* |  |  |  |
|  |  | *Age* | *-0.05* | *-0.15 – 0.05* |
|  |  | *Female* | *-0.19** | *-0.36 - -0.03* |
|  |  | *Ethnicity* | *-0.01* | *-0.22 – 0.19* |
|  |  | *Highest qualification* | *0.01* | *-0.06 – 0.08* |
|  |  | *Total household assets* | *0.07* | *-0.02 – 0.16* |
|  |  | *Household composition* | *0.02* | *-0.10 – 0.14* |
|  |  | *Partnered* | *-0.15* | *-0.36 – 0.07* |
|  |  | *Sexual touch* | *-0.08* | *-0.18 – 0.02* |
|  |  | *Alcohol consumption* | *-0.07* | *-0.19 – 0.06* |

*Note.* All coefficients are standardised, and 95% confidence intervals are reported. Negative *B* values indicate better health outcomes. Positive *B* values indicate worse health outcomes, suggesting more load on the respective system. *p < .05. **p < .01

**Table A.6.** *Cross-lagged path models predicting touch frequency with romantic partners and individual biomarkers for each latent health outcome (metabolic, cardiovascular, neuroendocrine)*

| **Final model** | **Romantic partner touch frequency** | |
| --- | --- | --- |
|  | *B* | 95% CI |
| **Metabolic** |  |  |
| Waist circumference (inches) | -.049 | -.11 - .01 |
| Log-transformed CRP (mg/L) | -.044 | -.14 - .05 |
| Log-transformed HbA1c (mmol/mol) | -.064 | -.15 - .03 |
| BMI (kg/m^2^) | **-.118**** | **-.19 - -.05** |
| **Cardiovascular** |  |  |
| sBP (mmHg) | **-.144*** | **-.26 - -.03** |
| dBP (mmHg) | **-.131*** | **-.23 - -.03** |
| **Neuroendocrine** |  |  |
| Pulse rate (beats per minute) | .053 | -.06 - .16 |
| DHEA (µmol/l) | .030 | -.09 - .15 |

*Note.* The final model is adjusted for sociodemographic characteristics (age, sex, ethnicity, educational attainment, and total household assets), household composition, romantic partner, sexual touch, and alcohol intake. Negative *B* values indicate better health, while positive *B* values indicate worse health, suggesting more load on the respective marker. **p* < .05. ***p* < .01

**Table A.7.** *Associations between romantic partner touch and subsequent health before and after adjustment for relationship happiness*

| Outcome | Model | β | SE | p | 95% CI |
| --- | --- | --- | --- | --- | --- |
| Metabolic health | Original | -0.078 | 0.0280 | .006 | -0.133 to -0.022 |
|  | + Relationship happiness | -0.084 | 0.030 | .005 | -0.142 to -0.026 |
| Cardiovascular health | Original | -0.137 | 0.048 | .005 | -0.232 to -0.042 |
|  | + Relationship happiness | -0.147 | 0.051 | .004 | -0.247 to -0.047 |
| Neuroendocrine health | Original | 0.040 | 0.041 | .327 | -0.040 to 0.121 |
|  | + Relationship happiness | 0.047 | 0.043 | .275 | -0.037 to 0.131 |

*Note.* Estimates are unstandardised regression coefficients. Models retained the covariates included in the final models of primary analyses.
